# Association between the morphokinetics of in-vitro-derived bovine embryos and the transcriptomic profile of the derived blastocysts

**DOI:** 10.1371/journal.pone.0276642

**Published:** 2022-10-26

**Authors:** Shira Yaacobi-Artzi, Dorit Kalo, Zvi Roth

**Affiliations:** Department of Animal Sciences, Robert H. Smith Faculty of Agriculture, Food and Environment, The Hebrew University, Rehovot, Israel; Justus Liebig Universitat Giessen, GERMANY

## Abstract

The time-lapse system is a non-invasive method that enables a continuous evaluation through embryo development. Here, we examined the association between the morphokinetics of the developing embryo and the transcriptomic profile of the formed blastocysts. Bovine oocytes were matured and fertilized in vitro; then, the putative zygotes were cultured in an incubator equipped with a time-lapse system. Based on the first-cleavage pattern, embryos were categorized as normal or abnormal (68.5±2.2 and 31.6±2.3%, respectively; *P*<0.001). A cleaved embryo was defined as normal when it first cleaved into two equal blastomeres; it was classified as synchronous or asynchronous according to its subsequent cleavages. An abnormal pattern was defined as direct, unequal, or reverse cleavage. Direct cleavage was classified as division from one cell directly into three or more blastomeres; unequal cleavage was classified as division that resulted in asymmetrically sized blastomeres; and reverse cleavage of the first division was classified as reduced number of blastomeres from two to one. Of the normally cleaving embryos, 60.2±3.1% underwent synchronous cleavage into 4, 8, and 16 blastomeres, and 39.7±3.1% cleaved asynchronously (*P*<0.001). The blastocyte formation rate was lower for the synchronously vs. the asynchronously cleaved embryos (*P<*0.03). The abnormally cleaved embryos showed low competence to develop to blastocysts, relative to the normally cleaved embryos (*P*<0.001). Microarray analysis revealed 895 and 643 differentially expressed genes in blastocysts that developed from synchronously and asynchronously cleaved embryos, respectively, relative to those that developed from directly cleaved embryos. The genes were related to the cell cycle, cell differentiation, metabolism, and apoptosis. About 180 differentially expressed genes were found between the synchronously vs. the asynchronously cleaved embryos, related to metabolism and the apoptosis mechanism. We provide the first evidence indicating that an embryo’s morphokinetics is associated with the transcriptome profile of the derived blastocyst, which might be practically relevant for the embryo transfer program.

## Introduction

Selecting embryos with high developmental competence is a prerequisite for successful pregnancy outcome after embryo transfer [1]. In practice, bovine blastocysts are selected shortly before their transfer, based on morphological criteria, according to the International Embryo Technology Society (IETS) guidelines [2]. Nevertheless, during early embryonic development, from the zygote to the blastocyst stage, the embryo undergoes dynamic and intensive morphological changes that determine its fate. Thus, utilizing static morphological criteria only at the blastocyst stage might be misleading and inadequate for quality evaluation of the embryo. Taken together, this approach is limited and subjective [3, 4]; therefore, a more reliable selection method is required.

To this end, a time-lapse system—a non-invasive automated tool—was modified to allow continuous monitoring of embryonic development without removing the culture dish from the incubator [5–9]. In human-assisted reproduction management, use of a time-lapse system enables defining several parameters to select embryos for transfer [10–13]. Among these parameters, the time of the first cleavage has been found to be a good parameter, since early-cleaving embryos are more likely than late-cleaving embryos to develop to the blastocyst stage [14, 15]. Moreover, in humans, the pregnancy rate, following the transfer of early-cleaved embryos, is higher than that of the late-cleaved embryos and is associated with reduced abortion events [16].

Similarly, previous studies in bovine, which were performed in conventional incubators, have been associated with the timing of the first zygotic cleavage into 2-cell stages with its competence to develop into a compact morula [17] or into a blastocyst [18–20]. Moreover, the timing of the first cleavage was associated with a high survival capacity following cryopreservation [18]. Other studies have used the time-lapse system to examine the embryo’s morphokinetics [9, 19–21]. Massip et al. [20] used the time-lapse system to examine the process of hatching in the bovine blastocyst and defined three patterns: (1) continuous expansion, which is followed by hatching; (2) discontinuous expansion; it is interrupted by a few contractions and thereafter, was followed by hatching; and (3) discontinuous expansion, interrupted by several rapid contractions, resulting in hatching failure. Using a logistic regression model, Sugimura et al. [9] examined the association between the developmental competence of bovine embryos and various morphokinetic parameters. These parameters included the timing of the first cleavage and the number of blastomeres at the end of the first cleavage. Somfai et al. [21] revealed that the lengths of the first and second cleavages were earlier in embryos that later developed to the blastocyst stage, relative to those that did not develop into blastocysts. A previous study suggested that the lag phase, a temporary developmental arrest between the fourth and the fifth division, is a good parameter to predict the development potential of an embryo [22]. The latter study reported that the probability of embryos to develop into a morula or blastocyst is higher when the lag phase began by starting at the eight-cell stage, compared with embryos with a small number of blastomeres. In support of this notion, a more recent study suggested that embryos with more than 6 blastomeres at the onset of the lag phase can be a good predictor of pregnancy success [9]. Nonetheless, more accurate morphokinetic parameters are required to predict the developmental potential of bovine embryos.

One of the big advantages of using a time-lapse system is being able to see the embryo cleavage patterns and identify the abnormal ones. Examples of abnormal patterns are (1) direct cleavage, in which a single blastomere cleaves into more than two daughter blastomeres [23], (2) unequal cleavage, i.e., producing asymmetrically sized blastomeres [8], and (3) reverse cleavage, expressed by reduced number of blastomeres, most likely due to blastomeres fusion or cytokinesis failure [24]. Based on studies in humans, these abnormal cleavage patterns are associated with reduced developmental competence and the quality of the formed embryo, i.e., its ability to be transferred, implanted, and to establish a pregnancy that will develop into healthy live offspring [12, 25, 26]. Moreover, previous studies in humans [23] and in bovine [9, 19, 21] have indicated that the directly cleaved embryo is associated with aneuploidy and chromosome abnormalities. In humans, the proportion of euploid blastocysts was relatively lower when the embryos underwent direct cleavage at the early stages of development [23]. Haploidy, polyploidy, and mixoploidy forms were recorded for bovine blastocysts that developed from directly cleaved embryos [9, 27]. In addition, the directly cleaved bovine embryos presented an increased proportion of aneuploid embryos and abnormal chromosome numbers [19, 21]. However, the molecular mechanisms underlying these alterations are not clear.

We previously reported that early-cleaving bovine embryos expressed higher developmental competence and a differential expression of *GDF9*, *POU5F1*, and *GAPDH* genes relative to their late-cleaving counterparts [28]. Milazzotto et al. [29] found that the differential expression of genes was related to embryonic metabolism in blastocysts derived from early- vs. late-cleaving embryos. In addition, a higher concentration of lipids was recorded in the blastocysts of the early-dividing group. Early cleaved bovine embryos, examined after 40, 112, and 186 h post-fertilization, expressed a different metabolomic pattern and transcript abundance compared with late-cleaved embryos [30]. Note, however, that previous studies [28–30] focused only on early- vs. late-cleaved embryos but not on the morphokinetics of further development stages. Moreover, to the best of our knowledge, there are no data that associate the morphokinetics of a bovine embryo through its first cleavages with the transcriptomic profile of blastocysts that developed. This was the main aim of the current study. Using both a time-lapse system and microarray analysis, we characterized differential gene expression in blastocysts that developed from synchronously, asynchronously, and directly cleaved embryos. These findings might have an added value when blastocysts are selected for embryo transfer programs.

## Materials and methods

### Experimental design

**Fig 1:**
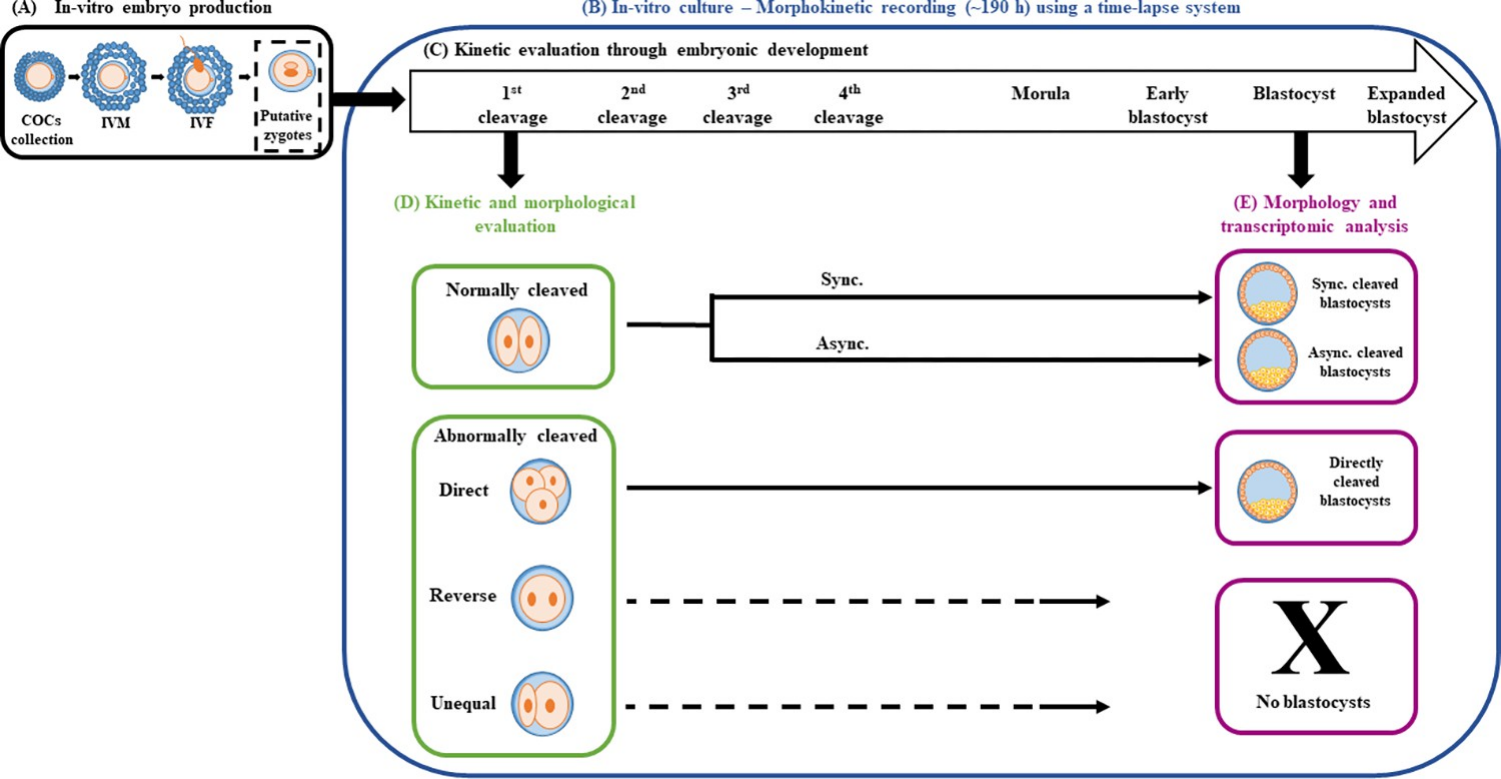
Experimental design. (A) Cumulus-oocyte complexes (COCs) were aspirated from bovine ovaries collected at the slaughterhouse and were in-vitro matured (IVM) and in-vitro fertilized (IVF). (B) Putative zygotes were individually cultured for 190 h in an incubator equipped with a time-lapse system to allow non-invasive, continuous monitoring of morphokinetic patterns throughout embryonic development. (C) Kinetics evaluation considered: (1) the timing of the 1^st^, 2^nd^, 3^rd^, and 4^th^ cleavages, morula formation, blastulation, blastocyst and expanded blastocyst formation and (2) the characterization of the cleavage pattern into synchronous (sync.; corresponding to cleavages to 4-, 8-, and 16-cell stage embryos) and asynchronous (async.; corresponding to cleavages to 3-, 5-, 6-, 7-, 10-, and 12-cell stage embryos). (D) Kinetics and morphological evaluation of embryos from the 1^st^ cleavage; characterization of normal or abnormal (direct, unequal, and reverse) cleavages and their morphological distribution into good, fair, and poor. (E) Morphology and transcriptomic evaluation of the developed blastocysts; blastocysts that developed from synchronously, asynchronously, and directly cleaved embryos were morphologically evaluated as good, fair, or poor. No blastocyst development was recorded for the unequally or reverse-cleaved embryos. Samples of 4 blastocysts from each of the three cleavage categories (synchronous, asynchronous, and direct) were subjected to RNA extraction, followed by microarray analysis, and samples of 3 blastocysts from each group were validated by RT-qPCR (4 replicates). Fig 1 was created by Shira Yaacobi-Artzi with Microsoft PowerPoint software.

The study was aimed at characterizing the morphokinetics of in-vitro-derived bovine embryos and further analyzing the gene expression profiles of the developed blastocysts (Fig 1(. The experiments were performed over 4 consecutive years, 2018–2021, during the winter to eliminate the effect of hot summer temperatures; they included 36 in-vitro production runs, 1,202 grade I oocytes, and 1,021 cleaved embryos (i.e., 2- to 4-cell stage embryos). For all in-vitro embryo production processes, oocytes were aspirated from ovaries collected from slaughterhouse post-mortem cows only; thus, living animals were not used in the experiments. The in-vitro model of bovine embryo production was approved by the Ethics Committee of the Hebrew University of Jerusalem (AG-22-16883-1). Cumulus-oocyte complexes (COCs) were collected, in-vitro matured, and fertilized (Fig 1A); putative zygotes were individually cultured for 190 h in an incubator equipped with a time-lapse system (Fig 1B). The timings of cleavages into 2, 4, 8, and 16 cells; morulas, early blastocysts, blastocysts, and expanded blastocysts were recorded (Fig 1C). The first cleavage patterns and morphologies were defined for each embryo (Fig 1D). The derived blastocysts were evaluated for their morphological appearance and subgroups were subjected to RNA extraction and microarray analysis (Fig 1E).

### Materials

All reagents were purchased from Merck-Sigma (Rehovot, Israel) unless otherwise specified. All culture media were prepared as previously reported [28, 31, 32]. This included HEPES–Tyrode’s lactate (HEPES-TL), which was supplemented with 0.3% (w/v) bovine serum albumin (BSA), 0.2 mM sodium pyruvate, and 0.75 mg/ml gentamicin (HEPES-TALP). Oocyte maturation medium (OMM) consisted of TCM-199 with Earle’s salts supplemented with 10% (v/v) heat-inactivated foetal bovine serum (Sartorius, Goettingen, Germany), 0.2 mM sodium pyruvate, 50 μg/μl gentamicin, 1.32 μg/ml porcine folltropin-V (Vetoquinol, Magny-Vernoi, France), and 2 μg/ml estradiol. Sperm-TL (SP-TL) was supplemented with 0.6% BSA, 1 mM sodium pyruvate, and 0.2 mg/ml gentamicin (SP-TALP). In-vitro fertilization-TL (IVF-TL) was supplemented with 0.6% essential fatty acid-free BSA, 0.2 mM sodium pyruvate, 0.05 mg/ml gentamicin, and 0.01 mg/ml heparin (IVF-TALP) as well as potassium simplex optimized medium (KSOM) for embryo culture that contained 95 mM NaCl, 2.5 mM KCl, 0.35 mM KH_2_PO_4_, 0.2 mM MgSO_4_·7H_2_O, 0.8% (v/v) sodium lactate, 0.2 mM sodium pyruvate, 0.2 mM d(+)-glucose, 25 mM NaHCO_3_, 1 mM l-glutamine, 0.01 mM Ethane-1,2-diyldinitrilo tetraacetic acid (EDTA), and 0.01 mM phenol red supplemented with 1.7 mM CaCl_2_·2H_2_O, 0.1 mg/ml polyvinyl alcohol, 10 μl/ml essential amino acids (Thermo Fisher Scientific, Waltha, USA), and 5 μl/ml non-essential amino acids (Thermo Fisher Scientific), as well as 100 U/ml penicillin-G and 0.1 mg/ml streptomycin.

### In-vitro embryo production

In-vitro production of embryos was performed as previously described [31, 33] with minor modifications. COCs were fertilized for 18 h with frozen spermatozoa of a single Holstein bull. At the end of fertilization, putative zygotes were carefully pipetted to remove remaining cumulus cells and adhering spermatozoa and were transferred individually (25 μl of KSOM, covered with mineral oil) into pre-equilibrated CultureCoin® (Esco Medical Group, Kringelled, Denmark), a culture dish with 14 microwells, specially designed for the Miri® TL time-lapse incubator from Esco Medical, corresponding to a total of 84 embryos. The CultureCoin dish was inserted into the Miri® Time-Lapse Incubator (MIRI® TLS, Esco Medical Group, Kringelled. Egaa, Denmark). The Miri® TL incubator includes 6 separate chambers for placing a maximum of 6 dishes of CultureCoin®. The built-in microscope was used to acquire images of each embryo every 5 min through seven focal planes, and images were taken by a built-in Zeiss objective (×20) with a numerical aperture of 0.35 specialized for 635 nm illumination (red light). All chambers were set up for the same culture condition, i.e., 38.5° C, 5% CO_2_, and 5% O_2_. (~190h) for each individual embryo. All videos were analyzed with Miri® TL Viewer software and assessed daily by the same person.

### Morphokinetics analysis

#### Embryonic developmental kinetics

The cleavage rates into 2- to 4-cell-stage embryos and the blastocyst formation rates were evaluated 42 h and ~190 h post-fertilization, respectively. The division times and the developmental stage times were defined as the time from fertilization (time 0), i.e., the initiation of incubation of the oocyte with the spermatozoa. Note that in the IVF procedure, the actual time of fertilization is unknown, and it might differ between individual oocytes. The embryonic developmental kinetics were monitored for each developmental stage. This included the intervals from fertilization to the first, second, and third divisions, while taking into account the number of cells in each embryo, i.e., 2, 3, 4, 5, 6, 7, 8, 10, 12, and 16 cells. Morulas were identified when embryo compaction was first observed, whereas blastomeres were still clearly distinguishable on the surface. The early blastocyst was recognized by the appearance of a stable cavity (blastocoel); the time at which the blastocyst started to expand was defined as blastocyst formation; the expanded blastocyst was characterized by an increase in the embryo diameter and a thin zona-pellucida, approximately one-third of its original size.

**Fig 2:**
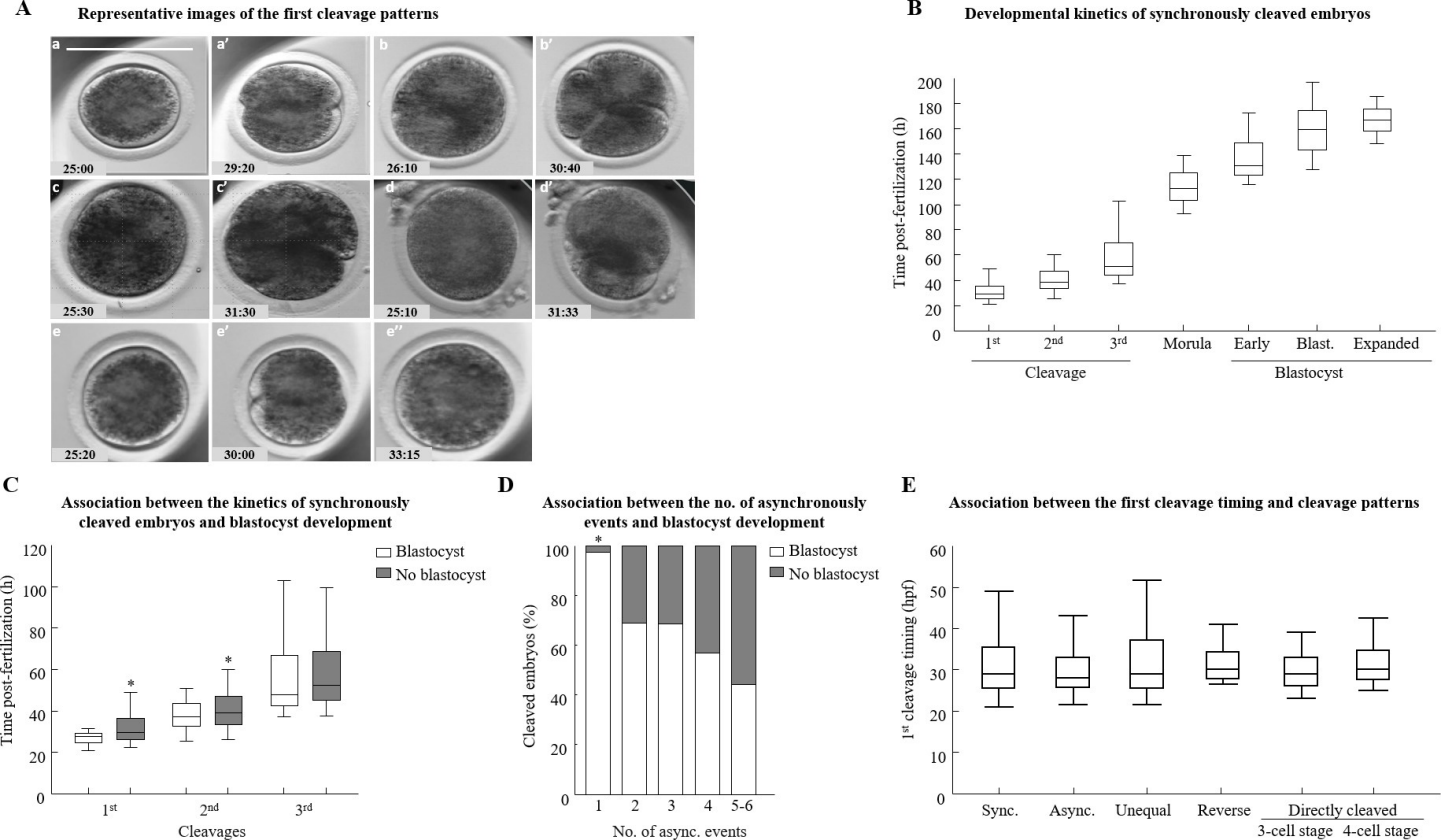
Developmental kinetics of cleaved bovine embryos. Cumulus-oocyte complexes (COCs) were in-vitro-matured, fertilized, and individually cultured for 190 h in CultureCoin dishes in an incubator equipped with a time-lapse system. Embryonic development was continuously monitored with image acquisition every 5 min. (A) Representative images of the morphological categories of embryos at the first cleavage: normally cleaved embryos are characterized by two equal-sized blastomeres (putative zygote after fertilization) (a), after normal cleavage (a’); directly cleaved embryos are characterized by direct cleavage from 1 cell (putative zygote after fertilization) to 3 blastomeres (b), after cleavage into a 3-cell-stage embryo (b’); or direct cleavage from 1 cell to 4 blastomeres (c and c’); an unequally cleaved embryo is characterized by two blastomeres of unequal size (d”); a reverse-cleaved embryo is characterized by reduced number of blastomeres through the first division (e and e’), and after merging to 1 cell (e”). The precise time (h:min) post-fertilization is presented in the lower-left corner of the pictures. Scale bar = 100 μm. (B) For each embryo in the synchronously cleaved embryos, the cleavage timing post-fertilization of the 1^st^, 2^nd^, and 3^rd^ cleavages, and the time of morula, early blastocyst, and blastocyst formation, as well as the blastocyst expansion were recorded. Data are presented in whisker plots. Boxes indicate the 25th and 75th percentiles, and the middle horizontal line indicates the median. Whiskers indicate the maximum and minimum values within the acceptable range defined by the two quartiles, n = 431 embryos. (C) Presented is the cleavage timing post-fertilization (1^st^, 2^nd^, and 3^rd^ cleavages) of synchronously cleaved embryos that further developed or did not develop to the blastocyst stage (the white or gray column, respectively). The line inside the box is the median; the lower hinge of the box indicates the 25th percentile, and the upper hinge indicates the 75th percentile. The upper whisker indicates the 95th percentile values and the lower whisker indicates the 10th percentile values for the cleaved embryos that further developed or did not develop to the blastocyst stage. The Kruskal Wallis test, followed by the Wilcoxon test pairwise comparisons, was used to compare the median value in the first, second, and third cleavages. *P < 0.05. (D) The pattern of asynchronous embryonic division was divided into six distinct classes, according to the number of asynchronous events expressed (i.e., one to six events). The proportion of embryos that either developed or did not develop to blastocysts was analyzed by the Chi-squared test was followed by Pearson’s test. *P < 0.05; n = 271 cleaved embryos. (E) Presented is the time of the first cleavage for all the cleavage patterns. The timing of the 1^st^ divisions in synchronously (Sync), asynchronously (Async), and abnormally cleaved embryos was recorded. The line inside the box is the median; the lower hinge of the box indicates the 25th percentile and the upper hinge indicates the 75th percentile. The upper whisker indicates the 95th percentile values and the lower whisker indicates the 10th percentile values. n = 656 embryos. The Kruskal Wallis test, followed by the Wilcoxon test, was used to compare the median value in the first cleavage between groups.

The embryo was defined as normally cleaved when cell cleavage resulted in two blastomeres of the same size (Fig 2A). Two subgroups of normally cleaved embryos were defined: synchronous and asynchronous. Synchronously cleaved embryos were characterized by synchronous divisions into 4, 8, and 16 blastomeres; asynchronously cleaved embryos were characterized by at least one asynchronous cleavage resulting in embryos with 3, 5, 6, 7, 10, or 12 blastomeres. In addition, some embryos exhibited more than 1 asynchronous cleavage event throughout their subsequent embryonic development. They were classified into 1, 2, 3, 4, or 5–6 classes, i.e., each class indicates the number of asynchronous cleavages. Since only a few embryos were classified with 5 or 6 asynchronous events, they were combined. Abnormally cleaved embryos included (1) directly cleaved embryos, i.e., direct cell cleavage from one blastomere to three or more blastomeres, (2) unequally cleaved embryos, i.e., the cell cleaved into two unevenly sized blastomeres, and (3) reverse-cleaved embryos, defined by reduced number of blastomeres from two to one through the first division (Fig 2A).

#### Embryo morphology through the first divisions

The normal synchronously cleaved embryos were morphologically evaluated at each developmental stage (i.e., 2-, 4-, 8-, and 16-cell stage) and scored as good, fair, or poor according to the IETS classification guidelines [2]. Good embryos had a symmetrical and spherical mass, blastomeres with uniform size, color and density, a smooth zona pellucida, and no concave or flat surfaces; fair embryos had moderately irregular blastomeres in shape, size, or color (at least 50% intact); poor embryos had blastomeres with major irregularities in shape or mass (at least 25% intact), size, or color.

The first-cleaved embryos of the asynchronous-, direct-, unequal-, and reverse-cleaved groups were also morphologically evaluated. The comparison of morphological distribution between the groups was based on the number of blastomeres presented: 2-cell synchronously cleaved vs. unequally and reverse-cleaved embryos; 3-cell asynchronously cleaved vs. 3-cell directly cleaved embryos; 4-cell synchronously cleaved vs. 4-cell directly cleaved embryos.

#### Blastocyst morphology

Blastocyst morphology was evaluated according to the IETS classification guidelines [2] with a few modifications. Morphology classification was based on the pattern and organization of the inner cell mass (ICM) and the trophoblast (TE) cells. The blastocyst’s morphology was defined as good when the ICM revealed many tightly packed cells and the TE cells had a cohesive epithelium; the embryo was defined as fair when the ICM exhibited a loose group of cells and the TE cells had a loose epithelium; the embryo was defined as poor when the ICM exhibited very few cells and the TE was composed of a few large cells.

### Transcriptomic analysis

#### Microarray analysis

Transcriptome analysis was performed on a pool of embryos that developed to the blastocyst stage, and all at the same developmental stage (n = 4 blastocysts per sample) taken from different IVF runs. Total RNA was extracted from blastocysts taken from asynchronously cleaved embryos (5 samples), synchronously cleaved embryos (3 samples), and from directly cleaved embryos (3 samples). Microarray analysis was performed as previously described by Komsky-Elbaz et al. [34]. Total RNA was isolated using the PicoPure RNA extraction kit (Arcturus, San Diego, CA), and purified using DNase I (Qiagen, Valencia, CA) at 25°C for 15 min. The total extracted RNA was stored at -80°C for further RNA labeling. Next, the RNA quality was analyzed by determining the RNA integrity number (RIN) using an Agilent 2100 Bioanalyzer (Palo Alto, CA) and only samples with RIN > 8.0 were used for the microarray analysis. The total RNA was amplified and labeled using the GeneChip WT Pico Reagent Kit (Affymetrix; Thermo Fisher Scientific, Waltham, MA), and then hybridized to the Affymetrix GeneChip Bovine Genome 1.0 ST Array (GPL16500). To prepare the hybridization probes, 400 pg of total RNA per sample were reverse-transcribed to cDNA using primers containing a T7 promoter sequence, so that the resulting cDNA contained the T7 sequence. Then the cDNA was amplified using low-cycle PCR, followed by linear amplification using T7 in-vitro transcription technology. The cDNA was converted to biotinylated sense-strand DNA-hybridization targets that were hybridized to the GeneChips in a GeneChip Hybridization Oven 640, using 1 chip per RNA sample, and then visualized using the Affymetrix GeneChip Scanner 3000. The original data were further processed using the Expression Console (V1.3.1.187; Affymetrix). Normalization, background subtraction, and a gene-level summary were performed using the Robust Multichip Average procedure with the default settings. The raw expression intensity values were determined with Transcriptome Analysis Console 4.0 (TAC4.0, Affymetrix) using ANOVA integrated into the software. The false discovery rate (FDR) procedure was also implemented in TAC4.0. The levels of significance were set to fold change > 2, *P* < 0.05 and FDR < 0.05. A principal component analysis (PCA), hierarchical clustering (unsupervised), and volcano plots were applied using the TAC 4.0 default settings; the distance is the Euclidean distance computed by the complete linkage method. Note that both the PCA and hierarchical clustering methods are considered complementary quality control, which emphasizes variations between samples. Volcano plots were also presented in order to show the statistical significance vs. the magnitude of change between groups.

#### Bioinformatics analysis

The differentially expressed genes detected by microarray analysis were deposited in the David functional annotation Bioinformatics Microarray Analysis tool (https://David.ncifcrf.gov/home.jsp) for gene ontology (GO) and biological function classification. In addition, the differentially expressed genes were input into the Search Tool for the Retrieval of Interacting Genes (STRING) database (https://string-db.org/), which demonstrates the biological relationship probabilities of gene-gene or the predicted protein–protein interactions.

#### Quantitative RT-PCR (RT-qPCR) analysis

Blastocysts from synchronously, asynchronously, and directly cleaved embryos were collected after 190 hours post-fertilization from four IVF runs for RT-qPCR assay to validate the microarray, as previously described [34, 35]. For each experimental group, 4 samples were used; each sample consisted of 3 blastocysts. Samples were washed in DNase-free PBS-PVP, snapped-frozen in liquid nitrogen, and stored at -80°C until RNA extraction. Poly(A) RNA was isolated using the Dynabeads mRNA DIRECT Kit according to the manufacturer’s instructions (Life Technologies, Carlsbad, CA, USA) as previously described [34, 35]. Briefly, blastocysts were lysed and mixed with prewashed Oligo (dT)_25_ Dynabeads. Following mRNA binding, each sample was washed twice with buffer A, twice with buffer B, and finally, mRNA was eluted with 10 mM Tris–HCl. The purified mRNA was used as the template in cDNA synthesis with SuperScript® III Reverse Transcriptase (Life Technologies) as previously described [35].

RT-qPCR was carried out with primers for 4 genes: *HSPA1A*, *ZP3*, *DDX3Y*, and GDF9 using *YWHAZ* and *SDHA* as the internal reference genes [32–35]. These genes of interest were selected from a list of differentially expressed genes (Table 1). These included overlapping genes between synchronously and asynchronously vs. directly cleaved embryos, Growth differentiation factor 9 (*GDF9)* and Zona Pellucida Glycoprotein 3 (*ZP3*); a non-overlapped gene between the synchronously vs. the directly cleaved embryos, DEAD-Box Helicase 3 Y-Linked (*DDX3Y)*, and differentially expressed genes in blastocysts from synchronously vs. asynchronously cleaved embryos, as well as Heat shock protein A1A (*HSPA1A*) and *DDX3Y*. Normalization for RT-qPCR was conducted using the geometric mean of two internal reference genes *YWHAZ* and *SDHA*. These genes were previously assessed and found to have stable expression in the blastocyst stage, independent of the experimental treatment [32–35]. The primers were derived from bovine sequences found in Genbank and specific primer pairs were designed using Primer 3.0 software. RT-qPCR was conducted using the LightCycler® 96 system (Roche, Basel, Switzerland) with the SYBR® Green qPCRBIO SyGreen Blue Mix Hi-ROX Kit (PCR Biosystems Ltd., London, UK) in a final volume of 20 μL containing ultrapure water (Biological Industries), 400 nM of each primer, and 3 μL diluted cDNA (1:4, v/v). A negative control, without reverse transcriptase, was included to ensure the absence of DNA template contamination. The reaction efficiency ranged between 90 and 110% with R2 > 0.995. The amplification program included preincubation at 95°C for 10 s to activate taq polymerase, followed by 40 amplification cycles of denaturation at 95°C for 10 s and annealing–elongation at 60°C for 15 s. All samples were run in duplicate The RT-qPCR data were analyzed according to the 2^-ΔΔCT^ method; they expressed the fold change of each selected gene within experimental groups. Data were normalized against the control (expression was set to 1). The fold change for each gene was subjected to one-way ANOVA followed by Student’s *t*-test.

**Table 1:** Primers used for qPCR analysis.

Gene	Gene Bank Accession number	Primer	Sequence (5’→3’)	Amplicon Size (bp)
*HSPA1A*	NM_203322.3	Forward	GCAGGTGTGTAACCCCATCA	181
		Reverse	CAGGGCAAGACCAAAGTCCA	
*ZP3*	NM_173974	Forward	GCCTGTTCCTTCAGCAAGTC	79
		Reverse	CCTTGCTACAGCATCGACAG	
*GDF9*	NM_174681	Forward	TGGTCCTTGCTGAAGCATCTAGA	202
		Reverse	ACAGTGTTGTAGAGGTGGCTTCT	
*DDX3Y*	NM_001172595	Forward	TCAGATGCTTGCTCGTGATT	120
		Reverse	GACCGTTTGTCTGCCTCTTC	
*YWHAZ*	NM_00174814	Forward	GCATCCCACAGACTATTTCC	124
		Reverse	GCAAAGACAATGACAGACCA	
*SDHA*	NM_174178	Forward	GGGAGGACTTCAAGGAGAGG	112
		Reverse	TCAACGTAGGAGAGCGTGTG	

### Statistical analysis

Data were analyzed by JMP Pro-15 software (SAS Institute Inc., 2004, Cary, NC). A comparison between cleavage and the blastocyst formation rates between the years 2018 and 2021 was subjected to one-way ANOVA, followed by the Tukey–Kramer test. However, before that, the proportion of cleavage to 2- to 4-cell-stage embryos and the blastocyst formation rate were arcsine transformed. Data are presented as the means ± SEM.

For every in-vitro production run, embryo developmental competence was analyzed by a one-way ANOVA followed by Student’s *t*-test or by the Tukey–Kramer test. Variables included the proportion of each pattern out of the total cleaved embryos, early blastocysts out of cleaved embryos, and blastocysts out of cleaved embryos. Student’s *t*-test was conducted for pair comparison between normal vs. abnormal and synchronously vs. asynchronously cleaved embryos. The Tukey–Kramer test was conducted for comparison between directly, unequally, and reversed cleaved embryos. All data were arcsine transformed before analysis. Data are presented as the means ± SEM.

A nominal logistic regression analysis was performed to evaluate the association between the first cleavage patterns (asynchronously, directly, unequally, and reverse cleaved embryos) and the blastocyst formation. Odd ratios and confidence intervals (CI) were calculated and normalized against the synchronously cleaved embryos. The Kruskal Wallis test, followed by the Wilcoxon test for pairwise comparisons, was used to compare the median time values of the first-, second-, and third cleavages within the synchronously cleaved embryos that either developed to blastocysts or did not, as well as for the first cleavage of synchronously, asynchronously, unequally, reverse, and directly cleaved embryos into 3- and 4-cell-stage embryos. Data for embryo kinetics are presented in box and whisker plots, indicating the timing for 25, 50 (i.e., median) and 75% of the cleaved embryos.

Correlation coefficients were calculated between the number of asynchronous events and their probability to develop into early blastocysts, as well as to the blastocyst stage.

An overall comparison of incidence data was performed by using Pearson’s chi-square test. Pairs of treatments were also compared by the chi-squared test, followed by Fisher’s exact test. Variables included the formation of blastocysts within the asynchronously cleaved embryo groups (corresponding to 1, 2, 3, 4, or 5–6 events) and the embryo morphology status for cleaved embryos and blastocysts.

For all analyses, *P <* 0.05 was considered significant. *P*-values of 0.05 and 0.1 were also reported as trends that might be realistic and worth noting.

## Results

### Cleavage patterns in embryonic development

The proportions of 2- to 4-cell stage embryos and of blastocysts were calculated out of the total cleaved embryos; they did not differ between the years, 2018, 2019, 2020, and 2021; therefore, the data were combined. The average proportion of 2- to 4-cell stage embryos was 85.4 ± 2.7% and the blastocyst formation rate was 26.4 ± 4.1%. It should be pointed out that the morphokinetic analysis (Table 2) included only embryos with an entire developmental MIRI® TLS record, i.e., from the zygote to the blastocyst stage. In case of partial or unclear records, embryos were excluded from the analysis. In addition, the developmental rate was calculated out of the cleaved embryos which enables to exclude some failure that associated with oocyte maturation, fertilization, or first divisions. Note that the main aim of the study was to examine the morphokinetic of early preimplantation embryos. Cleaved embryos (i.e., 2- to 4-cell stage embryos; n = 1021) were analyzed and defined as either normal (n = 702) or abnormal (n = 319), based on their cleavage pattern. The proportion of normally cleaved embryos was higher (*P* < 0.001) than that of the abnormally cleaved embryos (Table 2A). In addition, the proportion of embryos that further developed to the early or blastocyst stages was higher in the normal vs. the abnormal groups (*P* < 0.003 and *P* < 0.001, respectively; Table 2A).

**Table 2:** Embryonic development and blastocyst formation in normally and abnormally cleaving groups (2A) and subgroups (2B and 2C).

**2A**										
**Pattern**		**Normal first cleavage**			**Abnormal first cleavage**			***P value***		
Total cleaved embryos (n) Mean ± SEM %		702/1021 (68.5 ± 2.2)			319/1021 (31.6 ±2.3)			*P < 0*.*001*		
Total early blastocysts (n) Mean ± SEM %		211/702 (31.6 ± 3.1)			42/319(15.3 ± 2.9)			*P < 0*.*0003*		
Total blastocysts (n) Mean ± SEM %		146/702 (27.3 ± 3.4)			28/319 (7.9 ± 1.7)			*P < 0*.*001*		
**2B**										
**Pattern**			**Normal first cleavage**						***P value***	
**2A Subgroups**			**Synchronous**			**Asynchronous**				
Subgroup/total (n) Mean ± SEM %			431/702 (60.2 ± 3.1)			271/702 (39.7 ± 3.1)			*P < 0*.*001*	
Early blastocysts/cleaved embryos (n) Mean ± SEM %			94/431 (25.7 ± 3.9)			117/271 (50.6 ± 5.1)			*P < 0*.*0002*	
Blastocysts/cleaved embryos (n) Mean ± SEM %			65/431 (18.7 ± 3.7)			81/271 (31.8 ± 4.9)			*P < 0*.*03*	
**2C**										
**Pattern**	**Abnormal first cleavage**									***P value***
**Subgroups**	**Direct**			**Unequal**		**Reverse**	**More than one pattern**			
Subgroup/total (n) Mean ± SEM %	193/319 (66.8 ± 4.2)			95/319 (21.3 ± 4.07)		27/319 (10.9 ± 2.6)	4/319 (0.7 ± 0.4)			*P < 0*.*001*
Early blastocysts or cleaved embryos (n) Mean ± SEM %	35/193 (20.5 ± 4.6)			7/95 (5.8 ± 3.1)		0/27 (0)	*-*			*P < 0*.*001*
Blastocysts or cleaved embryos (n) Mean ± SEM %	24/193 (7.7 ± 3.1)			4/95 (4.2 ± 2.7)		0/27 (0)	*-*			*P = 0*.*1*

Among the normally cleaved embryos, there was a higher proportion of embryos that cleaved synchronously, compared with those cleaved asynchronously (*P* < 0.001). However, the proportion of cleaved embryos that developed to the early blastocyst stage was higher for the asynchronously vs. the synchronously cleaved group (*P* < 0.0002). In addition, the proportion of embryos that further developed to the blastocyst stage was higher in the asynchronously vs. the synchronously cleaved group (*P* < 0.03; Table 2B). Calculating the proportion of blastocysts that developed in the synchronously (n = 65) vs. the asynchronously (n = 81) cleaved subgroups, out of the total normally cleaved embryos (n = 702), indicated no differences between groups (10.2 ± 1.8 and 10.9 ± 1.9%, respectively).

Regarding the abnormally cleaved embryos, a higher proportion of embryos was found in the directly cleaved subgroup, compared with the unequally and reverse-cleaved subgroups (*P* < 0.001; Table 2C). Four embryos exhibited more than one abnormal event in the first cleavage (Table 2C). For example, unequal cleavage was followed by reverse cleavage. The proportion of early-stage blastocysts was higher for the direct-cleavage subgroup, compared with the unequal-cleavage subgroup (*P* < 0.001; Table 2C). The proportion of embryos that further developed to blastocysts did not differ between these subgroups (*P* = 0.1; Table 2C). No blastocyst development was recorded for the reverse-cleavage subgroup (Table 2C). Furthermore, the calculated proportion of blastocysts that developed from the directly cleaved embryos out of the total abnormally cleaved embryos tended to be higher (*P* < 0.09) relative to those that developed from unequally cleaved embryos (4.4 ± 1.5 vs. 1.8 ± 0.9%, respectively).

A logistic regression was conducted in order to examine a possible association between cleavage pattern and further embryonic development. The odds ratio of the blastocyst formation rate was 2.39 times higher in the asynchronously cleaved embryos than for the synchronously group (95% CI: 1.63–3.48; *P <* 0.0001). The odds ratios of the blastocyst formation rate for the directly and unequally cleaved embryos were 0.802 and 0.249 times lower than for the synchronously cleaved group (95% CI: 0.48–1.32; *P =* 0.38 and CI: -2.39–0.67; *P <* 0.003, respectively).

### Kinetic assessment

#### Kinetics of normally cleaved embryos and blastocyst formation

The developmental kinetics of synchronously cleaved embryos (n = 431) is presented in Fig 2B. For the synchronously cleaved embryos, the median time to 1^st^, 2^nd^, and 3^rd^ cleavage was 29.0, 38.7, and 51.0 h post-fertilization, respectively, and for morula, early blastocyst, blastocyst, and expanded blastocyst formation it was 113.0, 130.5, 159.5, and 166.5 h post-fertilization, respectively. Given that only a few embryos (n = 43) were clearly recorded at the 16-cell stage, data for this stage are not presented. To explore the differential morphokinetic patterns between normal synchronously cleaved embryos that developed to the blastocyst stage and those that did not, we examined the timing of the embryos’ first three cleavages. Embryos that developed to the blastocyst stage cleaved more rapidly through the first and second cleavages (*P* < 0.004 and *P* < 0.009, respectively; Fig 2C) and tended to be earlier for the third division, compared with those that did not develop to blastocysts (*P* < 0.08; Fig 2C).

The pattern of asynchronous embryonic division was divided into six distinct classes, according to the number of asynchronous events expressed (Fig 2D). The distribution of embryos that either developed or did not to the blastocyst stage differed between the five asynchronous classes (*P* < 0.002). In particular, a higher proportion of developed blastocysts was found in asynchronous embryos that underwent two, three, four, or five to six events of asynchronous cleavage, relative to those that had only one event (*P* < 0.002, *P* < 0.0005, *P* < 0.001 and *P*< 0.001, respectively; Fig 2D). In addition, the number of asynchronous events was highly correlated with development into early blastocysts and blastocyst stages (r = 0.8 and r = 0.9, respectively).

#### Kinetics of abnormally cleaved embryos and of blastocyst formation

A comparison of the cleavage timing between groups indicated that the time of the first cleavage for synchronously cleaved embryos did not differ from that of embryos that cleaved asynchronously or from embryos that directly cleaved to the 3-cell stage or to the 4-cell stage (Fig 2A and 2E). The timing of the first cleavage for synchronously cleaved embryos did not differ from that of the unequally or reverse cleaved embryos. Note that direct cleavage at the second and third division resulted in 8, 10, 12, or 16 cells; therefore, this could not be compared to the second and third divisions in the synchronously cleaved embryos, which resulted in 4 and 8 cells, respectively.

### Morphological assessment

#### Morphology of the cleaved embryos

**Fig 3:**
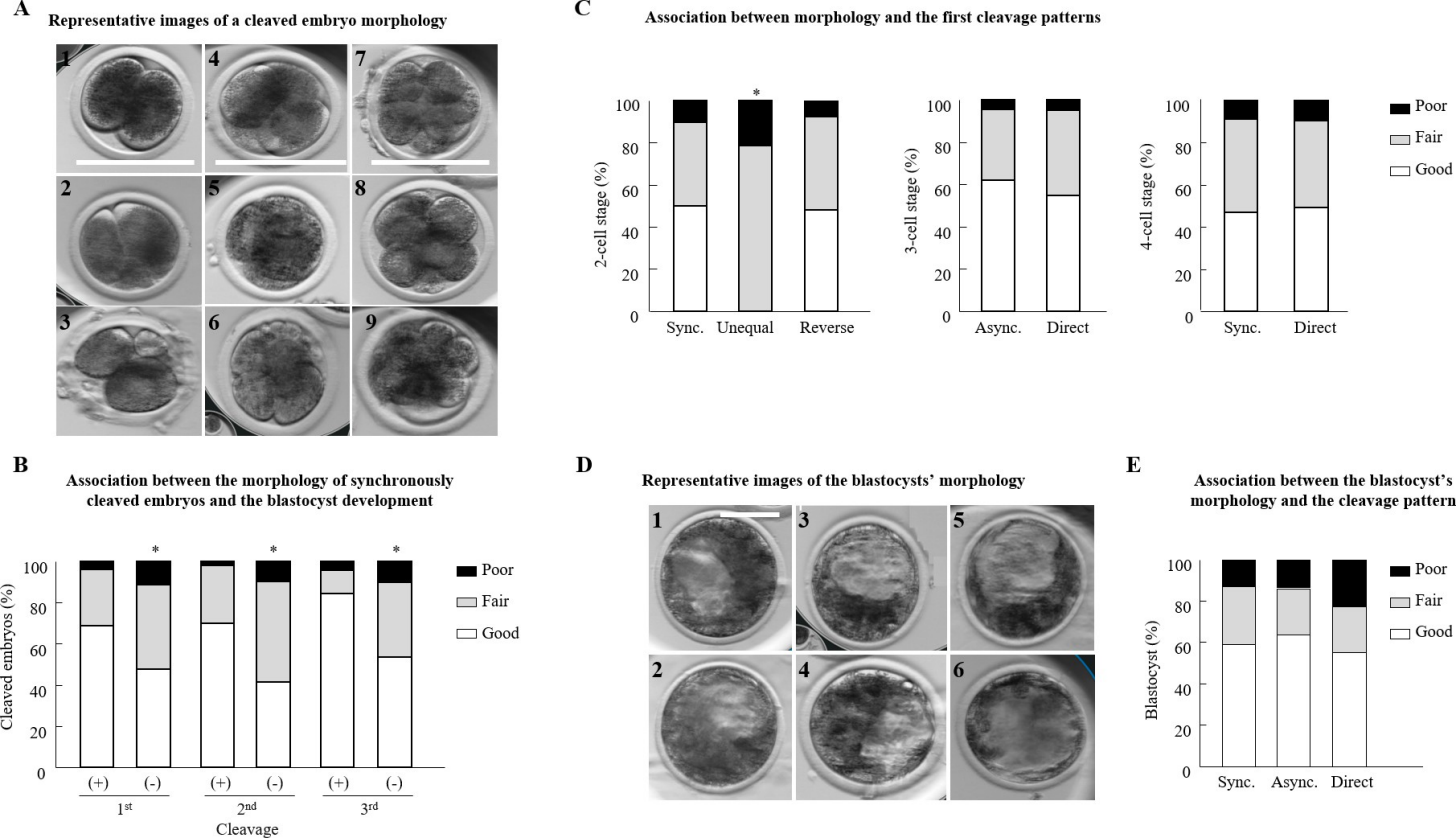
Morphology of bovine cleaved embryos. (A) Representative images of a 2-cell stage embryo with equal-sized blastomeres (1, good morphology); a 2-cell stage embryo with equal sized blastomeres and up to 20% fragmentation (2, fair morphology); a 2-cell stage embryo with unequal sized blastomeres and >50% fragmentation (3, poor morphology); a 4-cell stage embryo with four equal sized blastomeres (4, good morphology); a 4-cell stage embryo with fragmentation, equal sized blastomeres (5, fair morphology); a 4-cell stage embryo with unequal sized blastomeres and >50% fragmentation (6, poor morphology); an 8-cell stage embryo with equal sized blastomeres (7, good morphology); an 8-cell stage embryo with unequal sized blastomeres and minor (<20%) fragmentation (8, fair morphology); and an 8-cell stage embryo with unequal sized blastomeres and severe (>50%) fragmentation (9, poor morphology). (B) Distribution of normal synchronously cleaved embryos that developed (+) or did not (-) to the blastocyst stage into the different morphological categories (good, fair, and poor). A Chi-squared test, followed by Fisher’s exact test, was used for pair comparison within each cleavage. *P < 0.05; n = 431 embryos. (C) Distribution of 2-cell stage embryos with different cleavage patterns (synchronous; sync, unequal, and reverse) in different morphological categories. A Chi-squared test was followed by Pearson’s test; *P < 0.05; n = 403 embryos. Distribution of 3-cell stage embryos (from asynchronous and direct cleavage) in different morphological categories; n = 188 embryos. Distribution of 4-cell stage embryos (from synchronous and direct cleavage) in different morphological categories; n = 319 embryos. The distribution in morphological categories was calculated from cleaved embryos. A Chi-squared test, followed by Fisher’s exact test, was used. (D) Representative images of blastocysts with evident inner cell mass (ICM) with many tightly packed cells and many trophoblast (TE) cells forming a cohesive epithelium (1 and 2, good morphology); a blastocyst with a few TE cells forming a loose epithelium (3 and 4, fair morphology); a blastocyst with ICM presenting loosely grouped cells and TE composed of very few cells (5, fair morphology); a blastocyst with ICM presenting loosely grouped cells and TE composed of a few large cells (6, fair morphology), bar = 100 μm. (E) The distribution of synchronously (Sync), asynchronously (Async), and directly (Direct) cleaved embryos that developed to the blastocyst stage in the different morphological categories. A Chi-squared test, followed by Pearson’s test, was used, n = 170 blastocysts.

Embryo morphology was graded as good, fair, or poor (Fig 3A). The synchronously cleaved embryos that developed to the blastocyst stage differed in their morphology, as recorded in the first, second, and third cleavages, compared with those that did not develop to blastocysts (*P* < 0.001; Fig 3B). In particular, following the first cleavage, the distribution of embryos into morphological grades differed between groups (*P* < 0.01; Fig 3B), with a higher proportion of good-grade embryos among those that developed to blastocysts relative to those that did not (*P* < 0.006). Following the second cleavage, the distribution of embryos into morphological grades also differed between groups (*P <* 0.001; Fig 3B), expressed by a higher proportion of good-grade embryos (*P <* 0.0005) and a lower proportion of fair-grade embryos (*P* < 0.01) in those embryos that developed to blastocysts relative to those that did not. Similarly, following the third cleavage, the distribution of embryos into the three morphological grades differed between groups (*P* < 0.001; Fig 3B), expressed by a higher proportion of good-grade embryos (*P* = 0.0004) and a lower proportion of fair-grade embryos (*P* < 0.001; Fig 3B) in those that developed to blastocysts relative to those that did not.

#### Normally vs. abnormally cleaved embryos

The distribution of 2-cell stage embryos into morphological grades differed between synchronously and unequally cleaved embryos (*P* < 0.001; Fig 3C): the proportion of good grade embryos was higher in the former than in the latter (*P* < 0.001; Fig 3C). The proportion of fair grade embryos was lower in the synchronously vs. the unequally cleaved embryo groups (*P* < 0.001; Fig 3C). The proportion of poor grade embryos was lower in the synchronously cleaved embryos relative to the unequally cleaved embryos (*P* < 0.006; Fig 3C). The distribution of the reverse-cleaved embryos into the morphological grades did not differ from that of synchronously cleaved embryos (*P* = 0.8), but it did differ from that of the unequally cleaved embryos (*P* < 0.001; Fig 3C). This was reflected by (1) a higher proportion of good grade embryos (*P* < 0.001) and (2) a lower proportion of fair grade embryos (*P* < 0.006) in the reverse-cleaved vs. the unequally cleaved embryos. The distribution of asynchronously cleaved 3-cell stage embryos into morphological grades did not differ from that of the embryos directly cleaved into 3 cells (*P* = 0.6; Fig 3C). In addition, the distribution of embryos into morphological grades did not differ between normally cleaving embryos that cleaved through two synchronous cleavages into 4 cells, compared with those that cleaved directly into the 4-cell stage (*P =* 0.8; Fig 3C).

#### Blastocyst morphology

The morphology of blastocysts that developed from synchronously, asynchronously, and directly cleaved embryos was also defined as good, fair, or poor (Fig 3D). Only four of the unequally cleaved embryos developed to blastocysts and none of the reverse-cleaved embryos did. No difference was found between the distribution of the synchronous-, asynchronous-, and direct-cleavage blastocysts into morphological grades (*P* = 0.9; Fig 3E).

#### Transcriptome profiles

Blastocysts that developed from synchronously, asynchronously, and directly cleaved embryos were subjected to microarray analysis. A total of 24,415 genes were displayed using the bovine standard array. The findings indicated differential gene expression between blastocysts derived from the synchronous and asynchronous cleaved embryos and those derived from the directly cleaved embryos. Of the unequally or reverse-cleaved embryos, only a few developed into blastocysts; therefore, they were not included in the transcriptomic analysis.

**Fig 4:**
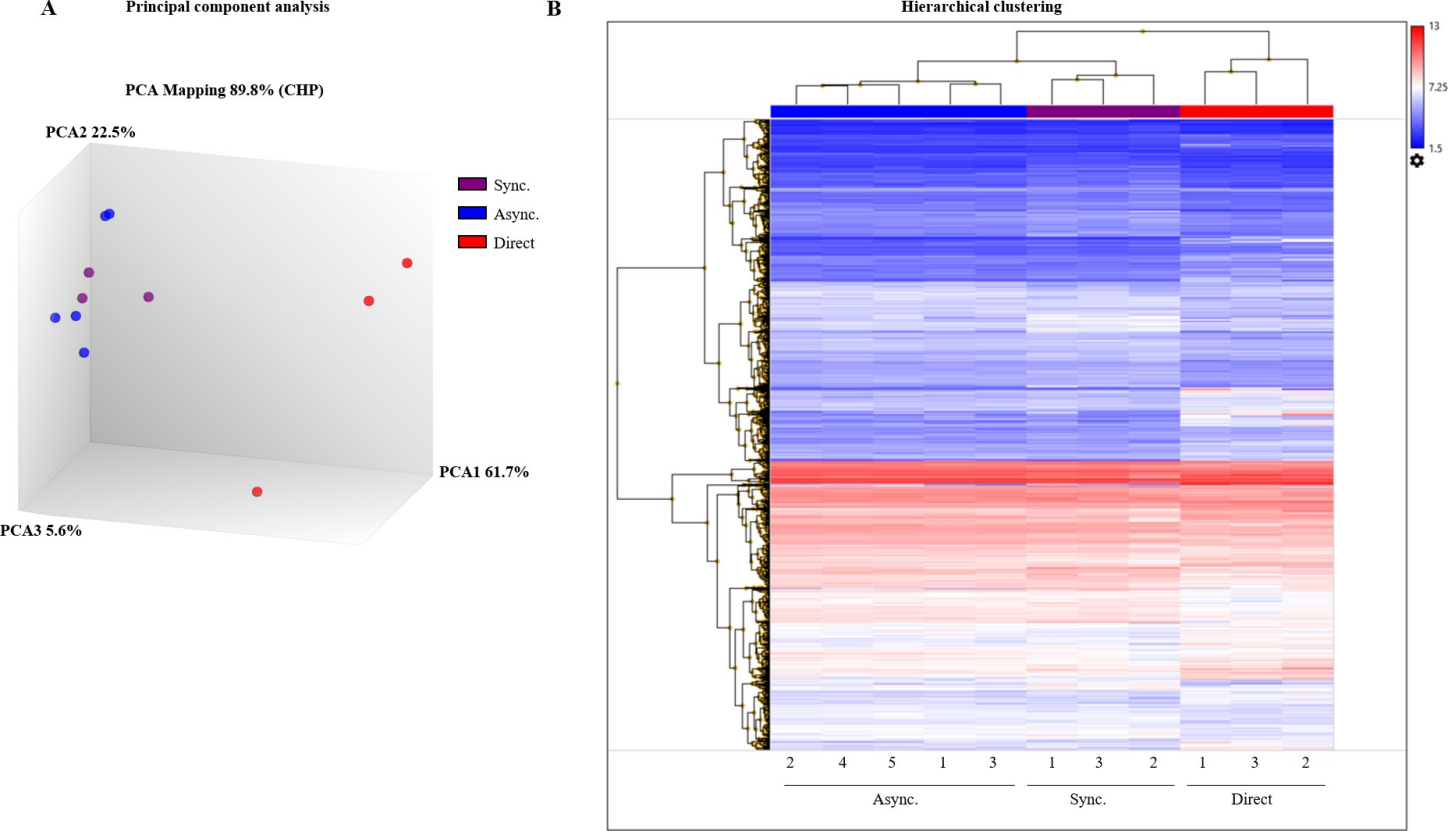
Differential gene expression in blastocysts that developed from synchronously, asynchronously, and directly cleaved embryos. (A) PCA plots. Purple, blastocysts from synchronously cleaved embryos (Sync); blue, from asynchronously cleaved embryos (Async); red, from directly cleaved embryos (Direct). Each symbol represents one replicate consisting of 4 blastocysts. (B) Hierarchical clustering (unsupervised) analysis of differentially expressed genes in blastocysts from synchronously, asynchronously, and directly cleaved embryos. Blastocysts from synchronously cleaved embryos are in 3 replicates (n = 4 blastocysts each) corresponding to samples Sync_1, Sync_3, and Sync_2; blastocysts from asynchronously cleaved embryos are in 5 replicates (n = 4 blastocysts each) corresponding to samples Async_1, Async_2, Async_3, Async_4, and Async_5; blastocysts from directly cleaved embryos are in 3 replicates (n = 4 blastocysts each) corresponding to samples Direct_1, Direct_3, and Direct_2. Colors indicate the expression levels of the detected genes: red, higher expressed; blue, lower expressed. Each horizontal line represents a single gene, and each column represents a single sample from each group. Left branches indicate the relationships among different genes and the upper branches indicate differences among samples.

The validity of the variation between groups was supported by PCA, which accounted for 89.8% of the variability. The x-axis of the PCA (PCA1) accounted for 61.7% of the variability between synchronously and asynchronously vs. the directly cleaved embryos (Fig 4A). Hierarchical clustering analysis was performed to better demonstrate the genes’ striking segregation into groups (Fig 4B). Blastocyst samples derived from asynchronously cleaved embryos shared the same branches as those from the synchronously cleaved embryos. However, they differed in their upper branches from the samples from the directly cleaved embryos.

#### The transcriptomic profile in blastocysts from normally cleaved embryos

**Fig 5:**
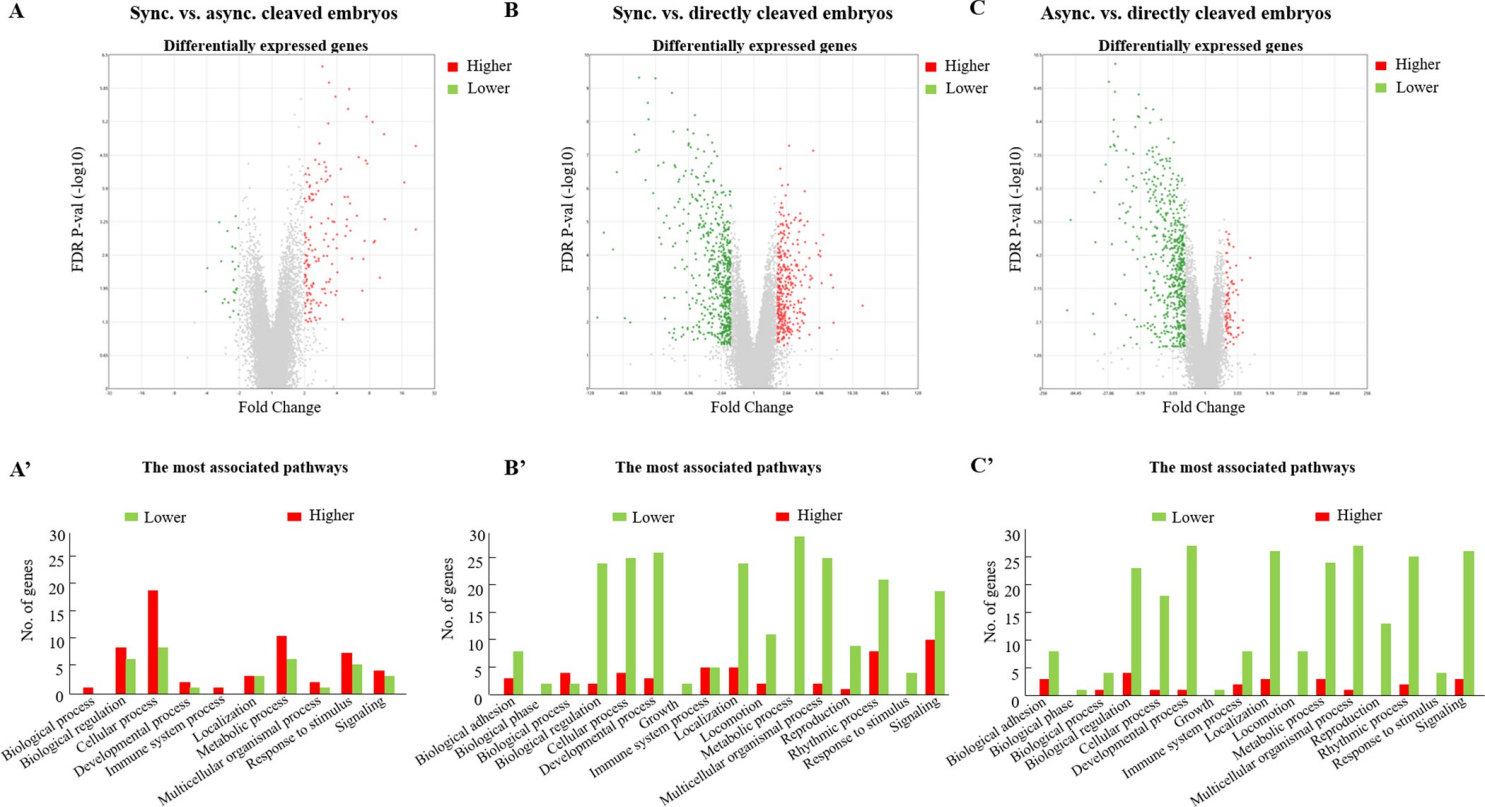
Differentially expressed genes between groups. Volcano plots are presented to visualize the differential gene expression patterns between groups. The values plotted on the x- and y-axes represent the average normalized signal values of each group (log2-scaled). For each gene, the *P*-value was plotted against the fold change. Vertical bars represent statistical significance and dots represent the genes that were higher expressed (red) or lower expressed (green). (A) Volcano plots present the differential gene expression between synchronously (sync) and asynchronously (async) cleaved embryos (n = 180 differentially expressed genes); (A’) Distribution of lower (green) and higher (red) differentially expressed genes within the most associated biological processes between synchronously vs. asynchronously cleaved embryos (B) A volcano plot presents the differential gene expression between synchronously and directly cleaved embryos (n = 895; differentially expressed genes); (B’) Distribution of lower (green) and higher (red) differentially expressed genes within the most associated biological processes and between synchronously vs. directly cleaved embryos. (C) A volcano plot presents the differential gene expression between asynchronously and directly cleaved embryos (n = 643; differentially expressed genes). (C’) The distribution of lower (green) and higher (red) differentially expressed genes within the most associated biological processes between synchronously vs. asynchronously cleaved embryos.

Differential expression of 180 genes was observed in blastocysts from synchronously vs. the asynchronously cleaved embryos (S1 File). Among these genes, the expression of 156 genes was higher, whereas the expression of 24 genes was lower in the synchronous, compared with the asynchronous group. A volcano plot was used to visualize this differential expression (Fig 5A and 5A’). Based on the DAVID functional annotation tool, 133 records were detected among the 180 differentially expressed genes. GO-enrichment analysis revealed that some of the differentially expressed genes were significantly (*P* < 0.05) enriched in biological processes and molecular function (Table 2). About 26 genes were associated with catalytic activity, 8 genes were associated with multiorganism processes, 4 genes were associated with the multiorganism reproductive process, and 4 genes were associated with sexual reproduction. Among them, *HBG* and *LOC788610* were involved in six different molecular functions: iron binding, heme, oxygen and tetrapyrrole binding, oxygen transport, and oxidoreductase activity. The expression of *HSPA1A* was higher in the synchronously vs. the asynchronously cleaved group. *HSPA1A* was involved in five biological processes: positive regulation of NF-kappaB transcription factor activity, multiorganism processes, sexual reproduction, the multiorganism reproductive process, and the positive regulation of sequence-specific DNA binding transcription factor activity. *HSPA1A* was also involved in one molecular function (catalytic activity). In addition, three genes were associated with biological processes and molecular functions. The expression of Glutathione S-transferase Mu 3) *GSTM3* (was higher in the blastocysts from synchronously vs. the asynchronously cleaved embryos. *GSTM3* was associated with molecular processes (glutathione transferase, catalytic activity, and transferase activity) and biological functions (the metabolic process and the glutathione metabolic process). The expression of *LOC785540* was higher, and that of *CYP51A1* was lower in blastocysts from synchronously vs. the asynchronously cleaved embryos. The latter was associated with the molecular processes of iron binding, heme binding, catalytic activity, tetrapyrrole binding, and oxidoreductase activity (Table 3).

**Table 3:** Enrichment analysis of differentially expressed genes in blastocysts that developed from synchronously vs. asynchronously cleaved embryos (DAVID).

Functional annotation	Gene count	Associated genes	Fisher exact test (*P-*value)
**Molecular function**			
Glutathione transferase	3	GDAP1↑; GSTM1↑; GSTM3↑	3.9E-06
Iron binding	5	LOC785540↑; CYP51A1↓; LOC783993↑; LOC788610↑; HBG↑	1.3E-04
Heme binding	4	LOC785540↑; CYP51A1↓; LOC788610↑; HBG↑	8.1E-04
Oxygen transporter activity	2	LOC788610↑; HBG↑	1.1E-03
Oxygen binding	2	LOC788610↑; HBG↑	1.6E-03
**Biological process**			
Metabolic process	2	GSTM1↑; GSTM3↑	3.5E-03
Gamete generation	1	SPIN2B↑	1.3E-03
Glutathione metabolic process	2	GDAP1↑; GSTM3↑	4.2E-03
Peptide catabolic process	2	CPQ↑; ERAP1↓	1.8E-03
Apoptosis positive regulation of NFkappaB transcription factor activity	3	CTH↓; EDA↑; HSPA1A↑	3.4E-03
**Biological process 1 (Goterm)**			
Multiorganism process	8	F2RL1↓; BMPR1B↑; CAV2↑; DAZL↑; HSPA1A↑; LYZ3↑; SLPI↑; SPIN2B↑	2.9E-02
**Biological process 2 (Goterm)**			
Sexual reproduction	4	BMPR1B↑; DAZL↑; HSPA1A↑; SPIN2B↑	1.9E-02
Multi-organism reproductive process	4	BMPR1B↑; DAZL↑; HSPA1A↑; SPIN2B↑	3.0E-02
Positive regulation of sequence-specific DNA binding transcription factor activity	3	CTH↓; EDA↑; HSPA1A↑	1.6E-02
**Molecular function 1 (Goterm)**			
Catalytic activity	26	HMGCS1↓; AGTPBP1↑; DDX3Y↓; WBSCR27↓; ALDH1A1↑;BMPR1B↓;	3.8E-03
		CPQ↑; CERS3↓; CTH↓;LOC785540↑; CYP51A1↓; ERAP1↓; EIF2S3Y↓;	
		FADS2↓; GDAP1↑; GSTM1↑; GSTM3↑; GZMB↑; HSPA1A↑;	
		LYZ3↑; NUDT10↑; PIGP↓; PCK2↓; SAT1↑; TUBA4A↑; LOC100125946↑	
**Molecular function 2 (Goterm)**			
Oxygen binding	2	LOC788610↑; HBG↑	1.6E-03
**Molecular function 3 (Goterm)**			
Transferase activity	4	CTH↓; GDAP1↑; GSTM1↑; GSTM3↑	1.9E-06
Tetrapyrrole binding	4	LOC785540↑; CYP51A1↑; LOC788610↑; HBG↑	1.1E-03
Oxygen transporter activity	2	LOC788610↑; HBG↑	1.1E-03
Oxidoreductase activity	3	LOC785540↑; CYP51A1↑; FADS2↓	1.5E-02

↑ higher expressed genes, ↓ lower expressed genes.

#### The transcriptomic profile of blastocysts from normally vs. abnormally cleaved embryos

The differential expression of 895 genes was observed between blastocysts from synchronously vs. directly cleaved embryos (*P* < 0.05; see the S1 File). A volcano plot was used to visualize this differential expression (Fig 5B). Among these genes, the expression of 330 genes was higher and that of 565 was lower in the former, compared with the latter (Fig 5B’). Comparing blastocysts derived from asynchronously vs. directly cleaved embryos revealed 643 differentially expressed genes (see the S1 File): the expression of 76 genes was higher and that of 567 genes was lower in the former, compared with the latter (Fig 5C and 5C’).

#### Overlapping genes in blastocysts from normally vs. abnormally cleaved embryos

**Fig 6:**
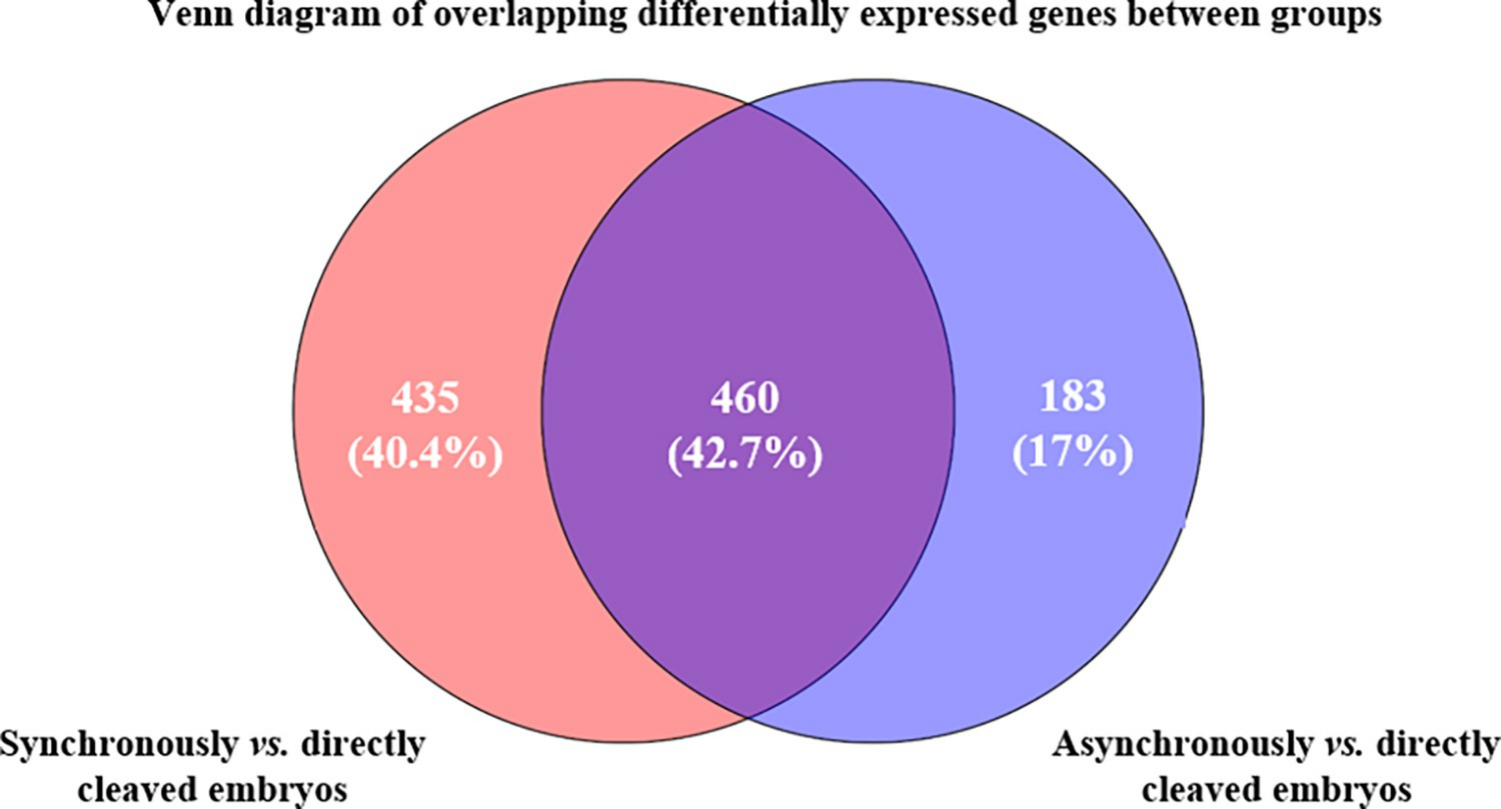
A Venn diagram of the differentially expressed genes (n = 1078 genes) in blastocysts derived from synchronously vs. directly cleaved embryos (pink, n = 435 genes); asynchronously vs. directly cleaved embryos (blue, n = 183 genes); and overlapping genes in blastocysts from synchronously and asynchronously vs. directly cleaved embryos (purple, n = 460 genes).

Venn analysis identified 460 genes that overlapped in blastocysts derived from synchronously and asynchronously cleaved embryos, where they were differentially expressed, compared with blastocysts derived from directly cleaved embryos (*P* < 0.05; Fig 6). Among the overlapping genes, 28 genes were highly expressed, and 380 genes were less expressed between the synchronous and asynchronous vs. the directly cleaved embryos, respectively. In addition, 4 genes were either highly expressed or less expressed in synchronous cleavage and asynchronous cleavage-derived blastocysts, compared with blastocysts derived from directly cleaved embryos (Fig 6). Note that 48 of the overlapping genes were not identified; therefore, they were not included in the analysis.

**Fig 7:**
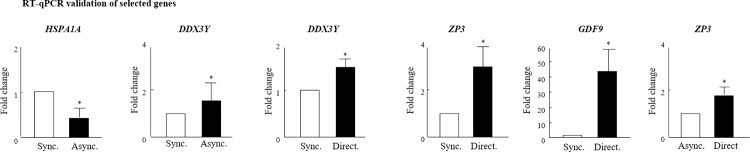
RT-qPCR validation of microarray analysis. Cumulus-oocyte complexes were in-vitro matured for 22 h and then fertilized. Putative zygotes were then in-vitro cultured for an additional 190 h to allow for blastocyst formation. Samples of the developed blastocysts (n = 3 for each sample; 4 replicates) from synchronously, asynchronously, and directly cleaved embryos were collected and their RNA was extracted for RT-qPCR validation based on four representative selected genes (*HSPA1A*, *ZP3*, *DDX3Y*, and *GDF9*). Presented is the fold change calculated by the 2^−ΔΔCT^ method, normalized against the reference genes *YWHAZ* and *SDHA* and relative to the control (synchronous or asynchronous, expressed as 1). Data are presented as the means ± SEM, *P < 0.05.

The microarray data were validated by RT-qPCR assay using four representative genes (*HSPA1A*, *ZP3*, *DDX3Y*, and *GDF9;* Fig 7). The relative expression of the four selected genes examined by RT-qPCR analysis (i.e., the fold difference in the expression of selected genes between experimental groups) was consistent with the profile obtained from the microarray analysis. For example, the fold change of the *HSPA1A gene was lower in the* asynchronously vs. the synchronously cleaved group *in both assays*, *exhibiting a fold change of 0*.*55 (P < 0*.*05) in the RT-qPCR and 2*.*14 (P < 0*.*0004) in the microarray*.

**Fig 8:**
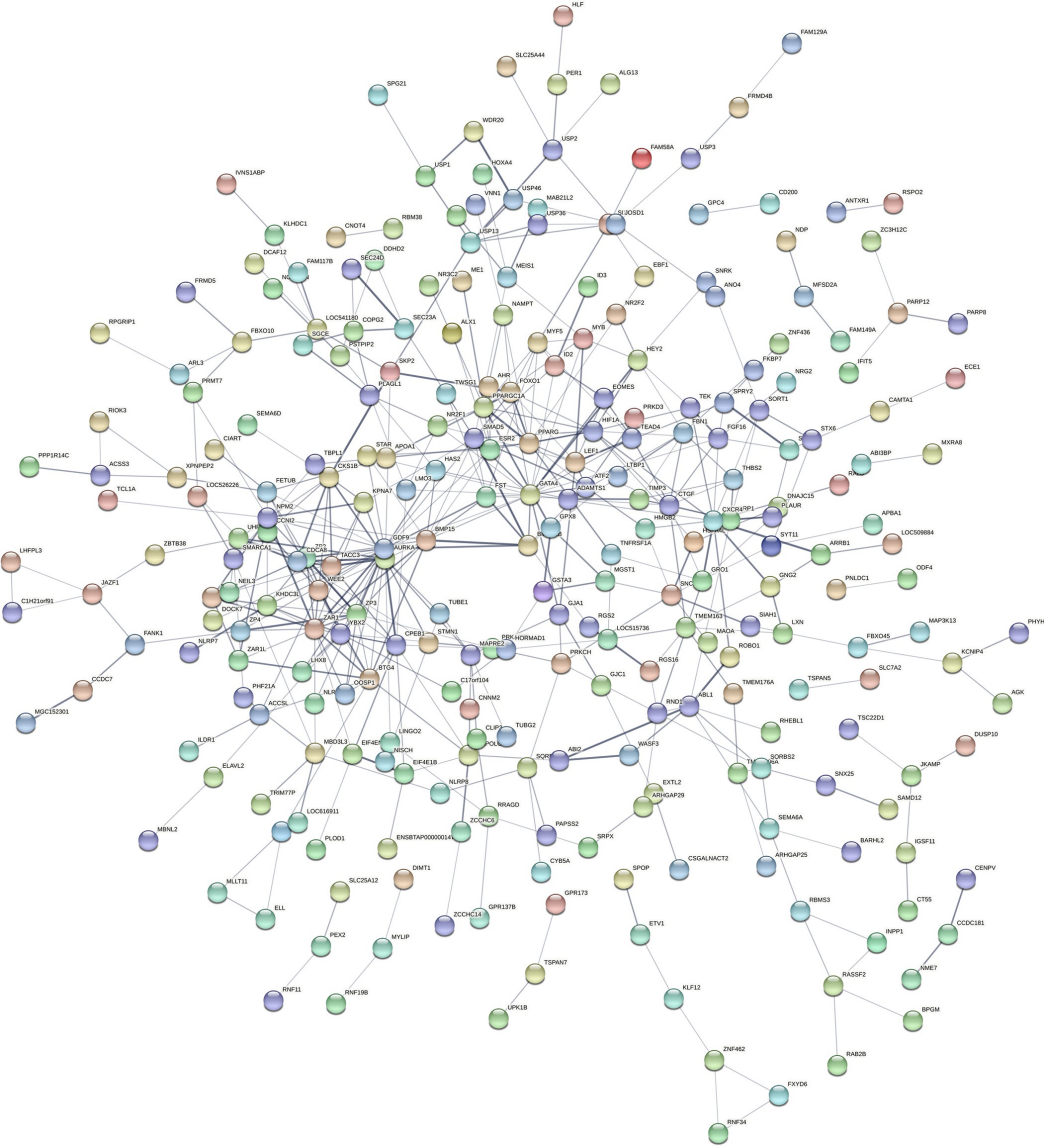
Possible protein–protein connections of overlapping differentially expressed genes. STRING analysis of the overlapping genes expressed differentially in blastocysts from synchronously and asynchronously vs. directly cleaved embryos. Only genes with nodes in the network association are presented. For a better view of the figure, it is highly recommended to use the magnifier tool.

STRING analysis was used to examine the biological relationship probabilities of gene-gene or the predicted protein–protein interactions between the overlapping genes. The differentially expressed genes were divided into four prominent networks (Fig 8). Key players (genes with more than 5 nodes) in one of the densest association networks were observed around the following genes: *GDF9* (18 nodes), *BMP15* (17 nodes), *FBN1* (6 nodes), *FST* (5 nodes), *LTBP1* (5 nodes), *ZAR1L* (14 nodes), *LTBP1* (5 nodes), *ZP2* (7 nodes), *FETUB* (6 nodes), *APOA1* (9 nodes), and *MXRA8* (7 nodes). Based on the DAVID analysis, *GDF9* and *BMP15* were differentially expressed in blastocysts from synchronously and asynchronously cleaved embryos, compared with those from directly cleaved embryos. These genes were directly linked to each other and significantly enriched in relation to cell development (Table 4). Another dense association network was observed around the following genes: *ZAR1* (14 nodes), *ZP3* (8 nodes), *ZP2* (8 nodes), *ZP4* (5 nodes), *NFM2* (7 nodes), *PAD16* (5 nodes), and *NPM2* (6 nodes). The expression of *NPM2*, which was enriched in the cellular component (nuclear chromatin), was lower in blastocysts from directly cleaved embryos. In addition, a dense association network was observed around the following genes: *AURKA* (11 nodes), *CKS1B* (8 nodes), *CDCA8* (5 nodes), *SKP2* (9 nodes), *UHRF1* (5 nodes), *TACC3* (6 nodes), *CPEB1* (10 nodes), and *WEE2* (8 nodes). According to the DAVID analysis, *AURKA* and *CDCA8* were significantly (*P* < 0.05) enriched in the cellular component of the spindle midzone (Table 4). In addition, *UHRF1* was significantly (*P* < 0.05) enriched in the cellular component of nuclear chromatin (Table 4). A fourth dense association network was observed around the following genes: *MYBLI* (5 nodes), *SIHA1* (5 nodes), *FBXO10* (7 nodes), *RNF19B* (6 nodes), *SKP2* (10 nodes), and *RNF34* (5 nodes).

**Table 4:** Enrichment analysis of overlapping genes in blastocysts that developed from synchronously and asynchronously cleaved embryos that are differentially expressed in directly cleaved embryos (DAVID).

Functional annotation	Gene count	Associated genes	Fisher exact test (*P*-value)
**Biological process**			
Cell differentiation	10	ABL1↓; ETV1↓; MYB↓;	2.5E-02
		PLAGL1↓; CTGF↑; FOXO1↓;	
		ODF4↓; PRKCH↓; TWSG1↓; USP42↓	
Apoptotic regulation	3	ABL1↓; TEK↓; SCG2↓	1.1E-02
Cell development	4	GATA4↓; BMP15↓; GJC1↓; GDF9↓	1.9E-02
Regulation of cyclin-dependent protein serine/threonine kinase activity	2	CCNB3↓; CCNI2↓	8.9E-03
Mitotic cell cycle phase transition		CCNB3↓; CCNI2↓; CKS1B↓	4.1E-04
**Cellular components**			
Cell cycle (Spindle midzone)	3	AURKA↓; CDCA8↓; CENPV↓	3.6E-02
(Nuclear chromatin)	7	SMARCA1↓; HMGB2 ↓; NPM2↓;	2.4E-02
		PLCB1↓; STAT1↓; UHRF1↓; USP3↓	
(Axonemal microtubule)	2	ARL6↓; SAXO1↓	1.2E-03
**Molecular function**			
Apoptotic process	4	ARRB1↑; LEF1↓; SNCA↓; MYB↓	1.1E-02
DNA binding	19	PPARGC1A↓; SMARCA1↓; TBPL1↓;	5.8E-02
		TEAD4↑; WDR76↓; YBX2↓; AHR↓;	
		BNC2↓; EBF1↓; LOC526226↓; HOMEZ↓;	
		HIF1A↓; MLF1↓; NOSTRIN↓; NR2F1↓;	
		NR2F2↓; PPARG↓; RNF141↓ STAT1↓	

↑ higher expressed genes, ↓ lower expressed genes relative to the blastocysts from synchronously cleaved embryos.

#### Non-overlapping genes in blastocysts from normally and abnormally cleaved embryos

A total of 435 and 183 non-overlapping differentially expressed genes were recorded between blastocysts from synchronously and asynchronously cleaved embryos and those from directly cleaved embryos, respectively (*P* < 0.05; Fig 6). Note that of the non-overlapping genes, 53 records were not identified; therefore, they were not included in the analysis.

A comparison of blastocysts from synchronously vs. directly cleaved embryos included 195 more highly expressed and 147 less expressed non-overlapping genes in the former. A comparison of blastocysts from asynchronously vs. directly cleaved embryos included 30 more highly expressed and 159 less expressed non-overlapping genes in the former.

**Fig 9:**
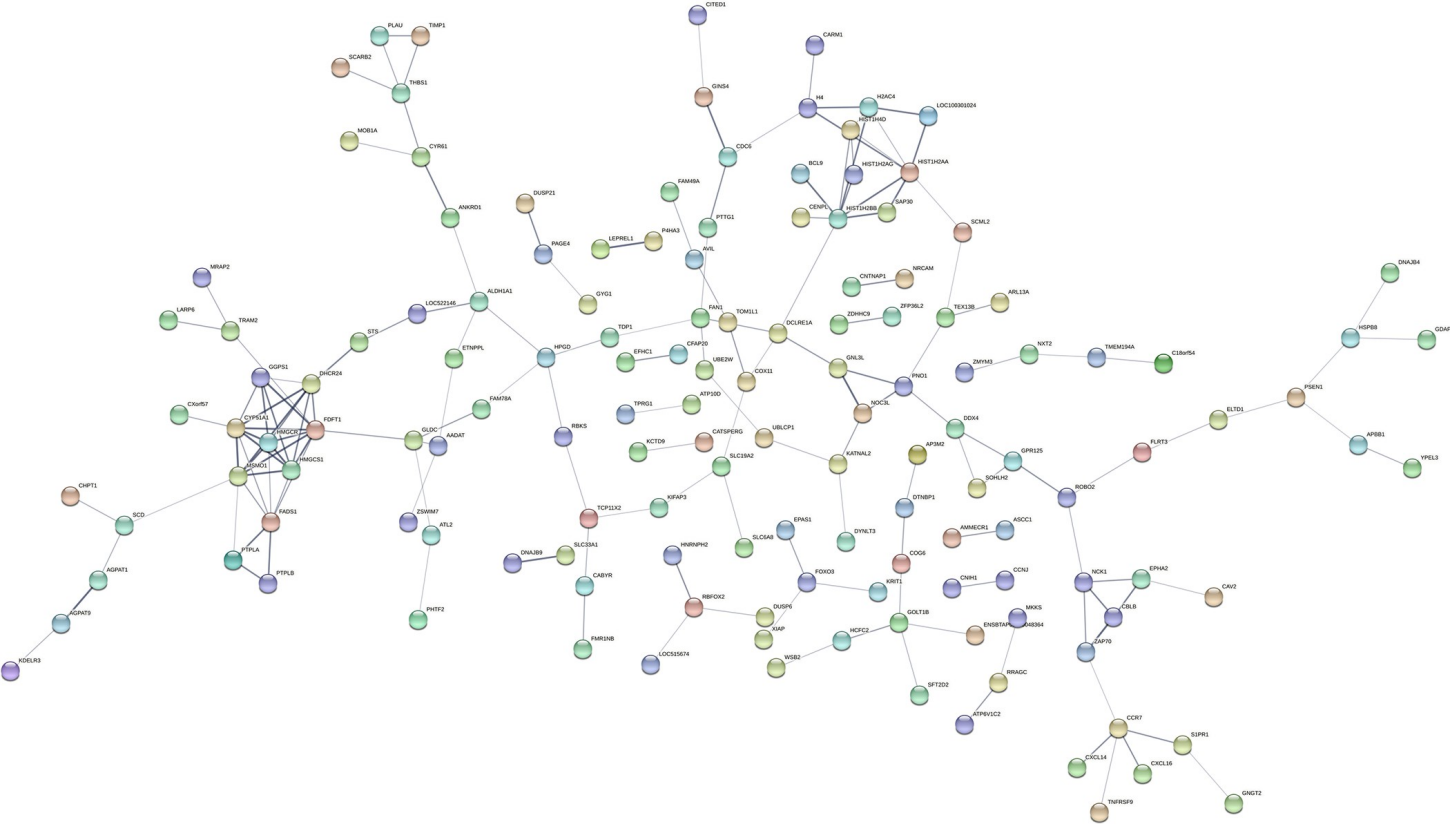
Possible protein–protein connections of non-overlapping differentially expressed genes. STRING analysis of non-overlapping genes expressed differentially in blastocysts from synchronously vs. directly cleaved embryos. Only genes with nodes in the network association are presented. For better view of the figure, it is highly recommended to use the magnifier tool.

**Fig 10:**
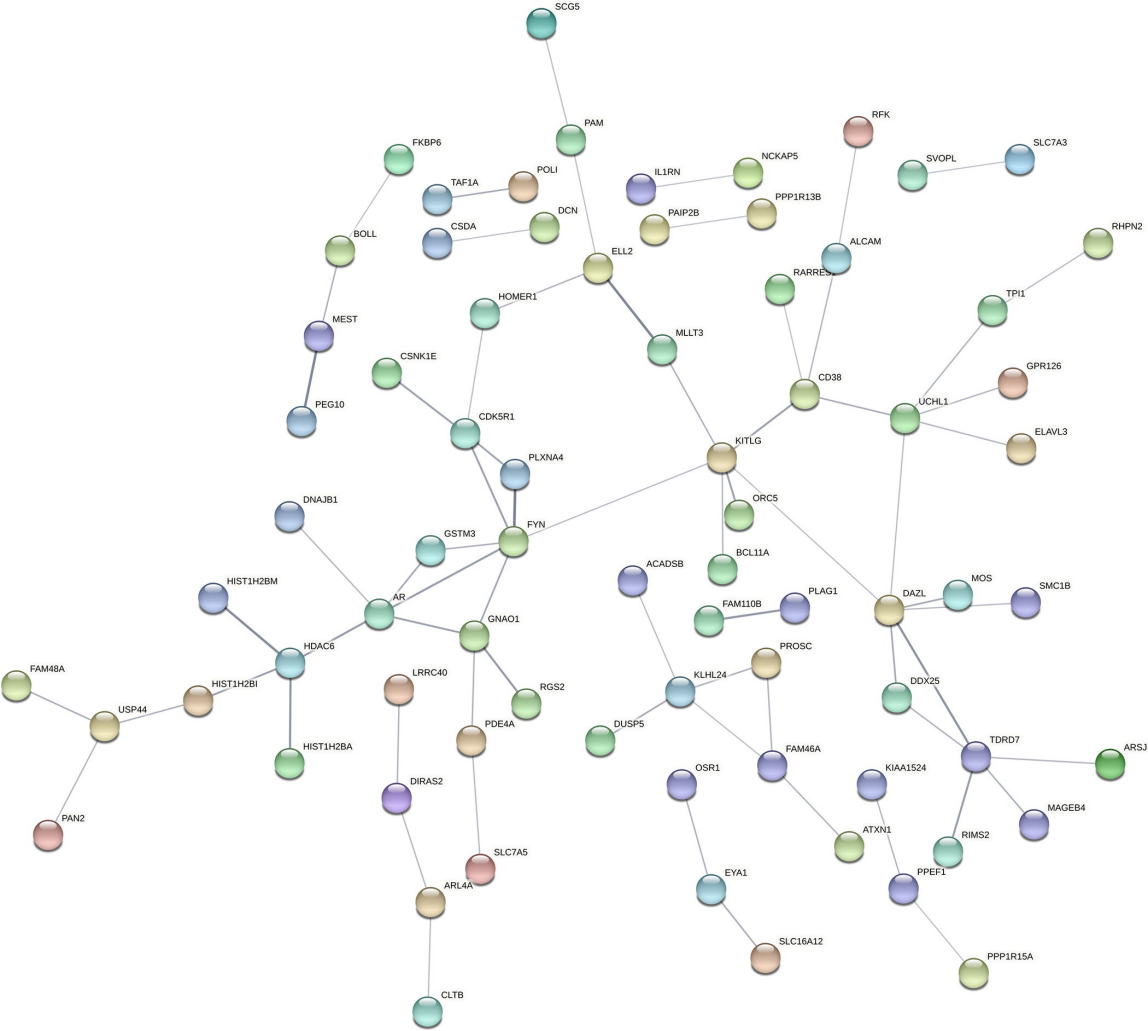
Possible protein–protein connections of non-overlapping differentially expressed genes. STRING analysis of non-overlapping genes expressed differentially in blastocysts derived from asynchronously vs. directly cleaved embryos. Only genes with nodes in the network association are presented. For a better view of the figure, it is highly recommended to use the magnifier tool.

Of the possible biological relationships of gene-gene or the predicted protein–protein interactions, two prominent networks were recorded for the differentially expressed genes in synchronous cleavage-derived blastocysts vs. direct cleavage-derived blastocysts (Fig 9). One of the densest association networks was observed around the following genes: DHCR24) 5 nodes), FDFT1) 8 nodes), *FADS1*) 5 nodes), *MSMO1*) 6 nodes), *CYP51A1*) 9 nodes), and *HMGCR* (8 nodes). GO-enrichment analysis revealed that *DHCR24*, *FDFT1*, *MSMO1*, and *CYP51A1* are enriched in molecular function or cellular components, based on the DAVID functional annotation tool (Table 5). Another dense association network was observed around the following genes: *CDC6*) 6 nodes), *HIST1H2BB*) 5 nodes), *BCL9*) 7 nodes), *HIST1H4D*) 6 nodes), and *CARM1* (7 nodes). The expression of all of these genes was lower in the blastocysts derived from synchronously vs. directly cleaved embryos. Two dense association networks were found for the differentially expressed genes in the asynchronous cleavage-derived blastocysts vs. the direct cleavage-derived ones (Fig 10). Among these genes, histone cluster 1, the H3i *HIST1H3I* gene, was enriched in nuclear nucleosome and *DAZL* was enriched in molecular function (Table 5).

**Table 5:** Enrichment analysis of non-overlapping genes in blastocysts that developed from synchronously or asynchronously cleaved embryos that are differentially expressed in directly cleaved embryos (David).

Group	Functional annotation	Gene count	Associated genes	Fisher exact test (*P*-value)
**Synchronous vs. direct cleavage groups**	**Biological process**			
	Mitochondrion organization	3	AGTPBP1↑; CAV2↑;	1.1E-02
			EPAS1↓	
	Response to oxidative stress	4	DHCR24↓; MTF1↓;	8.3E-03
			MSRB3↓; PSEN1↓	
	Apoptotic process	5	APBB1↑; ANKRD1↓; DUSP6↓;	3.1E-02
			PSEN1↓; ZNF346↓	
	Regulation of metabolic process	2	NPPC↑; THBS1↓	1.7E-03
	Regulation of cyclin-dependent protein serine/threonine kinase activity	2	CCNJ↓;	8.9E-03
	**Cellular components**			
	Endoplasmic reticulum	18	DHCR24↓; ATP10D↓; DNAJB9↓;	4.1E-02
			NCK1↓; ATL2↓; CERS3↓; CYP51A1↓; FDFT1↓; GOLT1B↓; KIFAP3↓; MRAP2↓; MSRB3↓; PSEN1↓; P3H2↓; PHTF2↓; RESP18↑; THBS1↓; ZDHHC9↑	
	**Molecular function**			
	Iron–iron binding	9	LOC785540↑; CYP51A1↓; LOC783993↑; LOC788610↑; HBG↑;	7.0E-05
			MSMO1↓; P3H2↓; P4HA3↓; SCD↓	
	Transcription factor binding	6	PPARGC1B↑; PIM1↓; RBFOX2↓; APBB1↑; STAT3↓; TRIB2↑	3.4E-04
	Ribonuclease-A activity	1	RNASE1↑	4.6E-04
**Asynchronous vs. direct cleavage groups**	**Biological process**			
	Regulation of cell differentiation	3	LOC100336475↓;	2.3E-05
			LOC101909114↓;	
			LOC100299732↓	
	Meiotic nuclear division	3	FKBP7↓; BOLL↓; SMC1B↓	2.7E-04
	Metabolic process	5	CD38↑; UGT1A1↑; UGT1A6↑;	1.7E-03
			ARSJ↓; GSTM3↓	
	Microtubule nucleation	1	CSNK1D↓	3.8E-03
	**Cellular components**			
	Nuclear nucleosome	3	LOC616868↓; HIST1H2BA↓; HIST1H3I↓	5.7E-03
	**Molecular function**			
	Protein serine kinase activity	6	CSNK1D↓; DCLK2↓; NRBP2↑;	2.6E-03
			STK38L↓; TRPM6↓; MOS↓	
	Translation activator activity	2	BOLL↓; DAZL↓	4.5E-04

↑ higher expressed genes, ↓ lower expressed genes

## Discussion

In the current study we associated the morphokinetic patterns of bovine embryo through the early cleavages with the transcriptome profiles of the blastocysts that developed. Morphokinetic analysis revealed that abnormally cleaved embryos exhibit a lower developmental competence than normally cleaved ones. Importantly, we provide the first evidence that blastocysts that developed from abnormal directly cleaved embryos differ in their transcriptomic profile from those stemming from normally cleaved embryos. We report here that blastocysts that developed from synchronously cleaved embryos differ in their transcriptome profile from those that developed from asynchronously cleaved embryos. It is worth noting that these two subgroups of embryos are considered normal and are routinely used in embryo transfer programs for both humans and animals.

### The timing of the three first cleavages is associated with the embryos’ developmental competence

Time-lapse systems are one of the most advanced techniques used in in-vitro embryo-production units. Continuous documentation of the developing embryos enables characterizing their morphokinetics without exposing them to disruptive conditions. In human IVF units, this approach is widely used to predict the developmental potential of individual embryos [11, 12]. On the other hand, in bovine, the time-lapse system is mostly used for research [9, 19, 21, 27]. Moreover, to date, the association between the embryo morphokinetics and the transcriptome profile of the developed bovine blastocyst has not been studied.

In humans, the timing of the first cleavage is generally accepted to be a good indicator of developmental competence [14, 15, 36]. The pregnancy rate, following the transfer of early-cleaved embryos, is relatively high and is associated with a reduced abortion rate [16]. Similarly, studies in bovine have shown that the timing of the first cleavage is a valuable parameter of embryo competence [17, 18, 37]. Here we report that not only the first cleavage, but also the three first embryonic cleavages are associated with the embryos’ competence to develop to the blastocyst stage. In support of this, Holm et al. [38] reported that in bovine, the first three cleavages occurred 1–2 h earlier in embryos that later developed to the morula or blastocyst stages, whereas the later-cleaved embryos were blocked at earlier developmental stages. Somfai et al. [26] showed that bovine embryos that developed to the blastocyst stage had shorter first and second cell cycles relative to embryos that did not develop into blastocysts. Similarly, a study in humans reported that earlier cleavage through the three first cleavages is associated with increased blastocyst formation [39]. Wong et al. [11] developed an algorithm, based on the duration of the three first divisions, to predict human blastocyst formation. It is therefore suggested that prediction of blastocyst formation, based on the first three cleavages, rather than on only the first cleavage, be utilized in bovine embryo transfer programs.

Selection of bovine embryos for transfer is largely based on morphological evaluation of the blastocyst shortly before transfer, a subjective approach that mostly relies on visual information at one particular stage [2, 9]. In humans, Conaghan et al. [40] suggested combining the time of the three first cleavages with the morphology of the 8-cell embryo on day 3 post-fertilization to select embryos with high developmental competence. In this regard, a deep learning method for bovine embryos, based on blastocyst morphology, has been suggested that takes into account continuous morphological evaluation [41, 42]. Here we report that embryos that expressed good morphology through the first three cleavages developed to the blastocyst stage, whereas embryos with a lower-grade morphology did not. Moreover, we suggest that morphological and kinetic parameters can be combined to predict the developmental potential of in-vitro-derived bovine embryos.

### Embryos with an abnormal cleavage pattern are of low developmental competence

In the current study we categorized embryos as being normally or abnormally cleaved. Our findings indicated that of the total cleaved embryos, the proportion of normally cleaved embryos was significantly higher than that of the abnormally cleaved ones. With respect to the abnormally cleaved embryos, we found a relatively high proportion of abnormally cleaved embryos (about 31.2%), and in particular, a high proportion (about 18.9%) of direct first cleavage. Interestingly, the timing of the first cleavage did not differ between directly, unequally, or reverse-cleaved embryos and that of normally cleaved embryos. In agreement with our findings, a previous study in bovine reported a high incidence of abnormal cleavage (46.9%) with 23.0% direct first cleavage [19]. In the same study, the time of the first cleavage was later for reverse- and direct-cleaved bovine embryos. Another study in humans reported that direct first cleavage occurs later than normal cleavage [43]. Nonetheless, whether the time of the first cleavage is a good parameter for identifying normal and abnormal embryos is not clear.

It is worth noting that embryos that develop from abnormal cleavage are of low developmental competence. In the current study, the occurrence of unequal and reverse cleavage was found to be determinate, since only 4 unequally cleaved embryos further developed to the blastocyst stage and no blastocysts developed from the reverse-cleaved embryos. In support of this, transfer of reverse-cleaved human embryos has been found to result in implantation failure [24]. Previous studies in humans suggest that reverse cleavage is associated with decreased cytoskeleton function, in particular, that of the actomyosin component, which is responsible for cytokinesis progression. Such an alteration might lead to polyploidy and chromosomal mosaicism or to an abnormality in the cell membrane [44]. Recent study in human, reported that about 25.9% of the developed blastocysts underwent reverse cleavage through the 3-first divisions. However, the incidence rate of reverse cleavage did not differ between euploid, mosaic and aneuploid, suggesting that reverse cleavage was not associated with the ploidy of the embryos [45]. Nevertheless, it should be pointed out, it is well accepted that the mechanisms that underlying the reverse cleavage involve blastomere fusion or cytokinesis failure [24].

Of the abnormally cleaved embryos, directly cleaved embryos warrant special attention because in the current study, about 12.4% of these embryos further developed to the blastocyst stage, and their distribution into morphological scoring did not differ from that of blastocysts that developed from normally cleaved embryos. Therefore, it reasonable to assume that in case of embryo selection that is based only on morphology, embryos from both groups will be considered suitable for transfer. In humans, directly cleaved embryos exhibit a low blastocyst formation rate [23] and reduced implantation competence [12, 23, 26, 46, 47]. Rubio et al. [43] observed that only 1.2% of directly cleaved embryos are implanted. In bovine, Magata et al. [19] reported that blastocysts developed from directly cleaved embryos present impaired hatchability with increased collapse of the blastocyst cavity until hatching. In contrast, Somfai et al. [21] reported that the potential of directly cleaved embryos to develop into blastocysts is similar to that of normally cleaved embryos. Sugimura et al. [8] reported that the potential of blastocysts that developed from directly cleaved embryos to establish pregnancy following embryo transfer is about 33.3% vs. 66.7% for normally cleaved embryos [8]. Taken together, both human and bovine studies provide clear evidence of the low competence of directly cleaved embryos to develop into blastocysts. Moreover, those that do develop to the blastocyst stage have low potential for transfer and pregnancy establishment.

### The pattern of first cleavages is associated with the transcriptomic profile of the derived blastocyst

The reduced developmental competence of directly cleaved embryos raises some questions about the mechanisms underlying implantation failure and pregnancy establishment. One possible explanation is genetic alterations. Our microarray analysis revealed differentially expressed genes in blastocysts that developed from directly cleaved embryos vs. those that developed from normally cleaved embryos. In particular, we identified a subgroup of genes that overlapped between blastocysts that developed from synchronously and asynchronously cleaved embryos vs. those derived from directly cleaved embryos. In addition, non-overlapping differentially expressed genes between synchronously and asynchronously cleaved embryos were also identified. These genes were involved in biological pathways, such as cell differentiation, cell division, metabolism, and apoptosis.

#### Cell cycle-associated genes

Previous studies in humans and bovine suggested that the phenomenon of directly cleaved embryos is associated with chromosome abnormalities [9, 19, 21, 23]. In humans, the proportion of euploid blastocysts was relatively lower when embryos underwent direct cleavage at early stages of development [23]. Directly cleaved bovine embryos presented an increased proportion of aneuploid embryos [19] or embryos with abnormal chromosome numbers [21]. In addition, haploidy, polyploidy, and mixoploidy forms were recorded for bovine blastocysts that developed from directly cleaved embryos [9]. Nonetheless, although the association between direct cleavage and chromosome abnormalities is well documented, the underlying mechanism is not clear enough. A previous comparison of the transcriptomic profile of human aneuploid vs. euploid embryos revealed that the enriched biological process was cell cycle regulation [48]. With that note, we report here that one of the enriched biological processes between the directly and normally cleaved embryos was associated with cell cycle mechanisms including regulation of cyclin-dependent protein serine/threonine kinase activity, mitotic cell cycle phase transition, and mitotic spindle organization. This was manifested by some differentially expressed genes that were highly expressed in the directly cleaved embryos relative to the normal cleaved ones. These genes included *AURKA*, *CDC6*, *CDCA8*, *CENPV*, *UHRF1*, *USP3*, *SMARCA1*, *HMGB2*, *PLCB1*, *STAT1*, *ARL6*, *SAXO1*, *CKS1B*, *CCNB3*, *CCNI2*, *CCNJ*, *PTTG1*, *SKP2*, *ABL1*, and *NPM2*. In support of this, according to STRING analysis, the densest association networks included the *AURKA*, *CKS1B*, *CDCA8*, and *UHRF1* genes. In mammals, Aurora A (AURKA) is involved in cell-cycle error-correction mechanisms through regulation of the spindle checkpoint [49, 50], thereby controlling meiotic events such as centrosome maturation, bipolar spindle assembly, and chromosome separation [51, 52]. Overexpression of *AURKA* has been shown to trigger genomic instability by dysregulating the normal steps of mitosis, potentially leading to aneuploidy [53], suggesting that a balanced level of *AURKA* is needed for proper embryonic development. In support of this, a higher expression of *AURKA* was also reported in human aneuploid cleaved embryos [47]. In addition, a higher expression of pituitary tumor-transforming gene 1 protein (*PTTG1)* was also found in directly cleaved embryos. *PTTG1* is a novel oncogene that is expressed abundantly in a variety of tumors [54]. A high expression of *PTTG1* in blastocysts is associated with no pregnancy and/or no calf delivery outcome [55, 56]. Taken together, it is possible that the higher expression of regulatory cell cycle genes in the directly cleaved embryos underlies their abnormal morphokinetic pattern and reduces their embryonic development.

#### Sex-associated genes

In searching for an additional mechanism that underlies morphokinetics, our microarray analysis provided slight evidence of a sex effect on embryo morphokinetics. Alteration in the expression of the *DDX3Y* and the Eukaryotic translation initiation factor 2 subunit 3 and the structural Y-linked *(EIF2S3Y)* genes, both located on the Y chromosome [57], were recorded. *DDX3Y* is restricted to male germ cells [58, 59] and *EIF2S3Y* was found to be exclusively expressed in male mouse blastocysts [60]. In accordance, the expression level of these genes is used for embryo sex determination, as was previously reported for bovine [57, 61, 62]. In the current study, although the timing of the first cleavage did not differ between synchronously and asynchronously cleaved embryos, the *DDX3Y* and *EIF2S3Y* genes were both highly expressed in blastocysts that developed from asynchronously cleaved embryos. On the other hand, the expression of the brain-expressed X-linked 2 *(BEX2)* and *HSPA1A* genes was higher in the synchronously vs. the asynchronously cleaved embryos. The *BEX2* and *HSPA1A* genes were previously found to be associated with the formation of female bovine blastocysts [63, 64]. In addition, blastocysts that developed from directly cleaved embryos were characterized by a higher expression of *DDX3Y* gene and a lower expression of *BEX2* relative to the synchronously cleaved embryos. Therefore, it is reasonable to assume that directly cleaved embryos with a higher expression of *DDX3Y* are male. Although not clear enough, the current study provides new evidence that the pattern of embryo cleavage, i.e., synchronously, asynchronously, and directly cleaved embryos are associated with the embryo sex. Nonetheless, findings that associate embryo sex with developmental kinetics are controversial. A previous study demonstrated that in-vitro-produced male bovine embryos were faster than female blastocysts, reflected by the fact that at day 7 post-insemination, 95% of the expanded blastocysts that developed were males [65]. Glucose supplementation into the culture medium affects the timing of the first cleavage of female and male bovine embryos: female bovine embryos were faster in the presence of glucose and male embryos were faster in its absence [66]. Other studies reported that embryo kinetic and blastocyst formation did not differ between male and female embryos [67–69]. Examination of embryo kinetics 24 to 48 h post-insemination [66] or 6 to 9 days post-insemination [67–69] did not reveal a difference between male and female bovine embryos. Another study on bovine embryos found no difference in the kinetics of the first cleavage between blastocysts that developed from X- and Y-sorted semen [67]. Taken together, it is suggested that morphokinetic patterns, in addition to the timing of the first cleavages, are sex related. Nonetheless, further studies are required to clarify this point.

#### Cell developmental regulation genes

We identified a subgroup of genes that overlapped between blastocysts that developed from synchronously and asynchronously cleaved embryos vs. those derived from directly cleaved embryos. Some of the overlapping genes were *GATA4*, *BMP15*, *ZP3*, and *GDF9*, which are associated with cell development. *GDF9* is a germ cell marker and a member of the large transforming growth factor-b superfamily. In mammals, *GDF9* is involved in the regulation of both oocyte and granulosa cell function at a very early stage of follicular development [70]. *GDF9* transcript is highly expressed in immature oocytes enclosed in the antral follicles, but it decreases throughout early embryonic development, with only trace levels at the 8-cell stage [71]. In the current study, results from both microarrays and RT-qPCR indicated a higher expression of *GDF9* in blastocysts that were derived from directly cleaved embryos, relative to those derived from synchronously and asynchronously cleaved embryos; both expressed an undetectable normal pattern. Gendelman and Roth [31] found a relatively high expression of *GDF9* in 4-cell stage bovine embryos that developed from oocytes that had been collected during the hot season, indicating a delayed decrease in *GDF9*, i.e., disruption in embryonic developmental competence during the hot season. Similar to *GDF9*, a higher expression level was also found for the *BMP15* gene in the directly cleaved embryos. BMP15 is known to be associated with follicular development and plays a fundamental role in oocyte development, ovulation, fertilization, and embryonic competence [72]. The expression level of *BMP15* is the highest at the metaphase II stage bovine oocyte, followed by a decrease until the 8-cell stage [73]. Therefore, a higher expression of *BMP15* in the directly cleaved embryos might be associated with disruption of cleavage. In support of this, a higher expression of *BMP15* in a 7-day blastocyst biopsy was associated with no calf delivery after embryo transfer [55].

Another group of genes that was found to be highly expressed in the directly cleaved embryo relative to the synchronously or asynchronously cleaved embryos belong to the zona pellucida (ZP) genes (*ZP2*, *ZP3*, and *ZP4)*. According to our GO analysis, these genes belong to the extracellular matrix, a cellular component that was found to be statistically enriched. The *ZP3* gene participates in the formation of the active ZP3/ZP4 complex that is responsible for sperm binding [74]. A previous bovine study suggested that these subgroups of genes are downregulated following fertilization [75]; however, this was not the case in the directly cleaved embryos. Taken together, the higher expression of *GDF9*, *BMP15*, *ZP2*, *ZP3*, and *ZP4* in blastocysts that developed from directly cleaved embryos might underlie, at least in part, the impaired first cleavage and the poor embryonic development recorded for this subgroup of embryos.

### Synchronously and asynchronously cleaved embryos differ in their transcriptomic profile

Studies in humans suggest that blastocysts that developed from synchronously and asynchronously cleaved embryos are both considered normal and suitable for transfer [76]. The current study provides new evidence that embryo competence to develop to the blastocyst stage differs between synchronously and asynchronously cleaved embryos. The proportion of synchronously cleaved embryos was higher than that of the asynchronously cleaved ones. However, embryos that underwent more than one asynchronous event during their development were associated with an increased probability to form a blastocyst. Although not definitive, there are a few reports in humans indicating that synchronously cleaved embryos are superior relative to asynchronously cleaved ones [77, 78]. For instance, one study showed that synchronously cleaved embryos have a higher survival rate, expressed by high-grade morphological embryos following cryopreservation, relative to the asynchronously cleaved ones. However, their capacity to be implanted, establish pregnancy, or result in birth did not differ between the subgroups [77]. On the other hand, another study in humans reported that the proportion of aneuploid embryos was higher in asynchronously cleaved vs. synchronously cleaved human embryos and concluded that the latter are more suitable for implantation than their asynchronously cleaved counterparts [78]. In agreement, Hardarson et al. [79] reported that asynchronously cleaved embryos with uneven cleavage or asymmetric blastomeres are associated with low pregnancy rates. In light of this, one might expect that similar to human asynchronously cleaved embryos, their bovine counterparts would express reduced developmental competence. However, this was not the case here; the proportion of asynchronously cleaved embryos that further developed to blastocysts was higher relative to that of the synchronously cleaved embryos.

Moreover, our microarray analysis provided the first evidence of a differential transcriptomic profile between synchronously and asynchronously cleaved embryos. For instance, the metabolic pathway was significantly enriched and included the glutathione S-transferase mu 1 (*GSTM1*) and *GSTM3* genes. These genes are involved in the detoxification of electrophilic compounds, such as oxygen radicals, via conjugation with glutathione [80]. In-vitro culture conditions, such as temperature, pH, and oxygen tension, have been reported to increase the reactive oxygen species and consequently, oxidative stress [81]. Therefore, the low expression of *GSTM1* and *GSTM3* found in blastocysts developed from asynchronously cleaved embryos might lead to an inferior antioxidative response upon exposure to oxidative stress in culture. Moreover, the low expression of *GSTM1* reported here for blastocysts from asynchronously cleaved embryos can result in modifications of the metabolic pathways that determine the glutathione level [82]. Women with *GSTM1* null polymorphism have an increased risk of recurrent pregnancy loss or a poor pregnancy outcome [83]. Taken together, one might expect that alterations in *GSTM1* and *GSTM3* expression might further affect the metabolic and oxidative status of the asynchronous embryos. This point should be studied further.

Differential expression in blastocysts from synchronously vs. asynchronously cleaved embryos was also recorded for three genes associated with apoptotic processes: *HSPA1A*, ectodysplasin A (*EDA*), and cystathionine-γ-lyase (*CTH*). Apoptosis can be defined as self-directed cell death that occurs to ensure normal development and differentiation of the embryo. In bovine, the apoptotic machinery appears at the 8- to 16-cell embryo stage [84], coinciding with embryonic genome activation. About 90 to 100% of morula- and blastocyst-stage embryos show apoptotic cells [85, 86]. *HSPA1A* participates in the apoptotic machinery by inhibiting mitochondrial depolarization by interrupting the BCL2 family signaling cascade, thereby inhibiting apoptosis [87]. A study of bovine preimplantation embryos reported that *HSPA1A* is differentially expressed throughout their development, suggesting that the apoptotic machinery is involved in the regulation of embryonic development [88]. Higher *HSPA1A* expression plays a critical role in the response to a variety of stressful environmental stimuli [89]. Here we report that *HSPA1A* expression was higher in blastocysts from synchronously relative to asynchronously cleaved embryos, which might suggest a higher competence to cope with stress and to survive cryopreservation and/or embryo transfer.

## Conclusion

The findings of the current study support the concept of using the morphokinetics of the embryo at early developmental stages to predict its developmental competence. The time-lapse system is a very useable tool, with safe and stable culture conditions. However, to date, this technique is not often used in bovine embryos due to the expensive equipment needed and because it is time consuming. Our study provides the first evidence that the bovine embryo’s morphokinetics is strongly associated with its developmental potential and is further expressed in the transcriptome profile of the resulting blastocyst. In particular, blastocysts that developed from directly cleaved embryos differed significantly in their gene expression profile from those stemming from normally cleaved embryos. Differential gene expression was also noted between synchronously and asynchronously cleaved embryos. Note, however, that the transcriptomic profile was conducted on a pool of blastocysts rather on a single embryo, which does not enable one to account for the potential differences in the embryo cell lineages (i.e., the pluripotent ICM and multipotent trophectoderm), as well as the differences between blastocyst developmental stages. In addition, given that the end point of the current study was blastocyst formation, the implications of the differentially expressed genes on further stages, such as implantation and post-implantation development outcomes were not examined. Nonetheless, our findings provide new evidence that morphokinetics at the early stages of embryo development is associated with the transcriptome profile of the formed blastocyst. These findings can contribute valuable information for embryo assessment prior to transferring the embryo.

## Supporting information

S1 File Raw data of the transcriptome analysis. (XLSX)
